# Scanning Photoelectrochemical Microscopy for the Investigation of Local Photocatalytic H_2_ Evolution in Matrixed Langmuir Films

**DOI:** 10.1021/acsami.5c26195

**Published:** 2026-05-01

**Authors:** Sarah Horn, Giada Caniglia, Sarah Jasmin Finkelmeyer, Gregor Neusser, Charlotte Mankel, Benedikt Bagemihl, Sven Rau, Christof Neumann, Andrey Turchanin, Riccarda Müller, Kerstin Leopold, Sidra Akther, Andrea Pannwitz, Stefan Zechel, Martin D. Hager, Ulrich S. Schubert, Moritz Jahn, Carsten Streb, Martin Presselt, Christine Kranz

**Affiliations:** † Institute of Analytical and Bioanalytical Chemistry, 9189 Ulm University, Albert-Einstein-Allee 11, Ulm 89081, Germany; ‡ Leibniz Institute of Photonic Technology (IPHT), Albert-Einstein-Str. 9, Jena 07745, Germany; § Laboratory of Organic and Macromolecular Chemistry (IOMC), Friedrich Schiller University Jena, Humboldtstr. 10, Jena 07743, Germany; ∥ Institute of Inorganic Chemistry I, Ulm University, Albert-Einstein-Allee 11, Ulm 89081, Germany; ⊥ Institute of Physical Chemistry and Abbe Center of Photonics, Friedrich Schiller University Jena, Lessingstrasse 10, Jena 07743, Germany; # Center for Energy and Environmental Chemistry Jena (CEEC Jena), Friedrich Schiller University Jena, Philosophenweg 7a, Jena 07743, Germany; ∇ Institute for Inorganic and Analytical Chemistry (IAAC), Friedrich Schiller University Jena, Humboldtstraße 8, Jena 07743, Germany; ○ Jena Center for Soft Matter (JCSM), Friedrich Schiller University Jena, Philosophenweg 7, Jena 07743, Germany; ◆ Helmholtz Institute for Polymers in Energy Applications Jena (HIPOLE Jena), Lessingstraße 12−14, Jena 07743, Germany; ¶ Helmholtz-Zentrum Berlin für Materialien und Energie, Hahn-Meitner-Platz 1, Berlin 14109, Germany; †† Department of Chemistry, 9182 Johannes Gutenberg University Mainz, Duesbergweg 10-14, Mainz 55128, Germany; ‡‡ Sciclus GmbH & Co. KG, Moritz-von-Rohr-Str. 1a, Jena 07745, Germany

**Keywords:** Langmuir films, photocatalysis, hydrogen evolution, scanning electrochemical microscopy, local illumination

## Abstract

Molecule-in-matrix systems such as Langmuir films with photosensitizers and hydrogen evolution reaction (HER) catalysts may exhibit heterogeneity in photocatalytic activity across large-area films. Here, we introduce a confined illumination approach and simultaneous localized detection of light-driven hydrogen (H_2_) evolution to directly correlate information on structural organization with catalytic performance of Langmuir–Blodgett and Langmuir–Schaefer films with incorporated **[Mo**_**3**_ **S**_**13**_ **]** ^**2–**^ catalyst and a Ru-based photosensitizer. Derived from a coating of optical fibers used in scanning near-field optical microscopy (SNOM), the outside of the glass sheath of the microelectrode was modified with a silver coating. Coupling light into the coated glass sheath, local illumination with suppressed light loss, and visible light excitation directly beneath the microsensor apex could be achieved, resulting in a 3.4-fold higher photon flux density compared to the uncovered microelectrode. Using platinum-black-modified microelectrodes as sensitive H_2_ microsensors, a bifunctional probe serving simultaneously as a local light waveguide and an H_2_ microsensor, enables quantitative detection of photogenerated H_2_ with spatial resolution. This is demonstrated here for films comprising a phospholipid matrix or a π-conjugated rigid molecular scaffold coembedding Ru-based photosensitizers and **[Mo**_**3**_ **S**_**13**_ **]** ^**2–**^ catalysts. This enables not only the local quantification of H_2_, but also the determination of the apparent quantum efficiencies (AQEs) of the different film architectures.

## Graphical abstract

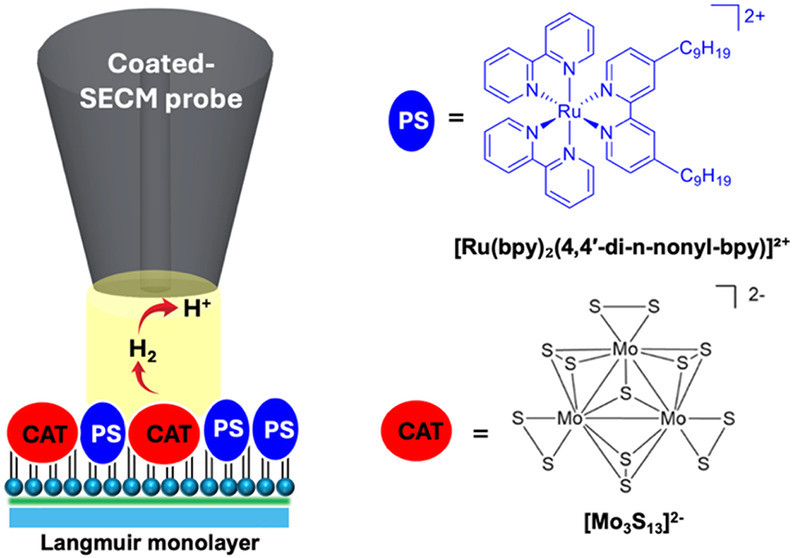

## 1 Introduction

Meeting the growing global energy demand requires the development of sustainable and carbon-neutral ways to produce, e.g., hydrogen. Using sunlight and water, artificial photosynthesis aims to mimic the natural photosynthetic process, in which complex photoactive molecular architectures embedded within transmembrane protein structures of thylakoid membranes drive light-induced redox reactions to convert solar energy into chemical fuels.^1,2^ In analogy, artificial systems integrate photosensitizers (PSs) and catalysts (CATs) within soft or hybrid matrices, such as nanoporous block copolymers,^3,4^ hydrogels,^5^ and lipid-based assemblies^6–8^ to enable light-driven H_2_ evolution, water oxidation (O_2_ evolution), or carbon dioxide (CO_2_) reduction.^5,6,8,9^ Among molecular PSs, ruthenium­(II) polypyridyl complexes remain the benchmark due to their long-lived excited states and tunable structural and redox properties.^10,11^ As molecular CATs for the H_2_ evolution reaction (HER), thiomolybdate clusters, especially **[Mo**_**3**_ **S**_**13**_ **]** ^**2–**^, are well-established non-noble metal alternatives,^12–14^ owing to their structural stability and excellent performance. Among the different molybdenum sulfide species, the thiomolybdate **[Mo**_**3**_ **S**_**13**_ **]** ^**2–**^ has shown excellent catalytic activity in homogeneous light-driven HER, with turnover numbers (TON) of tens of thousands.^13^ Inspired by the natural soft-matter integration of photocatalytically active components embedding PS and CAT into, e.g., lipid bilayers and vesicles has proven to be a very successful strategy.^6^ To improve access to the catalytically active sites of the immobilized PS and CAT, two-dimensional assemblies such as Langmuir monolayers at the air–water interface, formed by an amphiphilic matrix, are attractive to embed PS and CAT for more controlled integration. Langmuir–Blodgett (LB)^15,16^ and Langmuir–Schaefer (LS)^17^ deposition techniques lead to the controlled transfer of such molecular monolayers from the air–water interface onto solid supports. The Langmuir monolayer approaches have been applied to amphiphiles,^18^ polymers,^19,20^ biomolecules,^21,22^ dyes,^23,24^ electron acceptors,^25^ and photocatalytic systems.^26,27^ However, achieving uniform photocatalytic activity across large-area films remains challenging, as the film quality strongly depends on parameters such as compression, surface pressure, pH, and transfer speed.^28–30^ LS transfer can improve the coverage homogeneity compared to LB,^20,31^ while additives such as stearic acid (**SA**) have been shown to enhance their mechanical stability.^32^ Bulk measurement techniques such as gas chromatography fail to resolve local variations in photocatalytic activity, which can arise from inhomogeneous PS and CAT distributions or defects within the transferred films.^6,33^ Scanning electrochemical microscopy (SECM)^34,35^ provides an elegant way to probe such systems, typically with lateral resolution in the micrometer range,^36,37^ although nanoelectrodes have been reported to provide nanoscale resolution.^38,39^ Spatially resolved detection of reaction products such as H_2_ or O_2_ using microsized electrodes^40^ or microsensors^41^ is usually obtained in substrate generation/tip collection (SG/TC) mode.^42^ Yet, most of the localized measurements to date employ bulk illumination, where the entire film is illuminated, and most PS molecules of a large area are excited,^43^ leading to product accumulation in the bulk and possible degradation (photobleaching) of the PS, limiting the advantages of local measurements. To overcome this limitation, scanning photoelectrochemical microscopy (SPECM) has been introduced, which combines localized light excitation with an electrochemical readout. Early approaches used metal-coated optical fibers as a combined light source and electrode^43–45^ to study, for example, the oxygen evolution reaction on BiVO_4_ photoanodes.^45,46^ More recent designs couple an optical fiber to the glass sheath of a micro- or nanoelectrode,^39,47–49^ which serves as a waveguide to locally illuminate individual areas of a sample or single entities such as photocatalytically active particles.^39,49^ However, such configurations still suffer from significant light losses due to imperfect alignment and losses through the glass interface.^48,49^

Here, we introduce a bifunctional microelectrode that enables the electrochemical detection of formed products while the spatially confined measurement spot is illuminated. The glass sheath of the microelectrode is modified with an opaque layer, e.g., a mirror-like silver layer deposited via the Tollens reaction.^50^ This reflective sheath confines light propagation and minimizes losses, producing a sharply focused illumination spot directly below the electrode apex. By coupling this coating with Pt-black-modification of the electroactive area,^33^ we obtain a bifunctional SECM probe for localized illumination and H_2_ measurement, enabling quantitative detection of photogenerated H_2_ at a defined distance above the photocatalytic surface. This configuration allows local photocatalytic measurements with optical confinement at the measurement spot. Therefore, light-driven activity measurements can be performed at various spots of the same macroscopic sample, avoiding possible problems associated with the photobleaching of large sample areas.

## 2 Results and Discussion

### 2.1 Fabrication and Characterization of SPECM Microsensors

To improve the size of the illumination spot and the light intensity at the surface, a silver mirror coating was selectively deposited on the outer surface of the glass sheath of the microelectrodes, as illustrated in Figure 1a. Silver was chosen due to its high optical reflectivity across the visible range of the electromagnetic spectrum (>95 %) and low absorption losses.^51,52^ The purpose of the coating was to minimize stray light and improve the photon flux. The silver layer was applied using the classical Tollens reaction,^50^ which relies on the reduction of a diamminesilver­(I) complex using a mild reducing agent (glucose) to deposit elemental silver. Specifically, the Tollens reagent is formed in situ by forming the diamminesilver­(I) complex [Ag­(NH_3_)_2_]^+^. Upon addition of glucose, the silver ions are reduced to metallic Ag^0^ while glucose is oxidized to gluconolactone.

**Figure 1:**
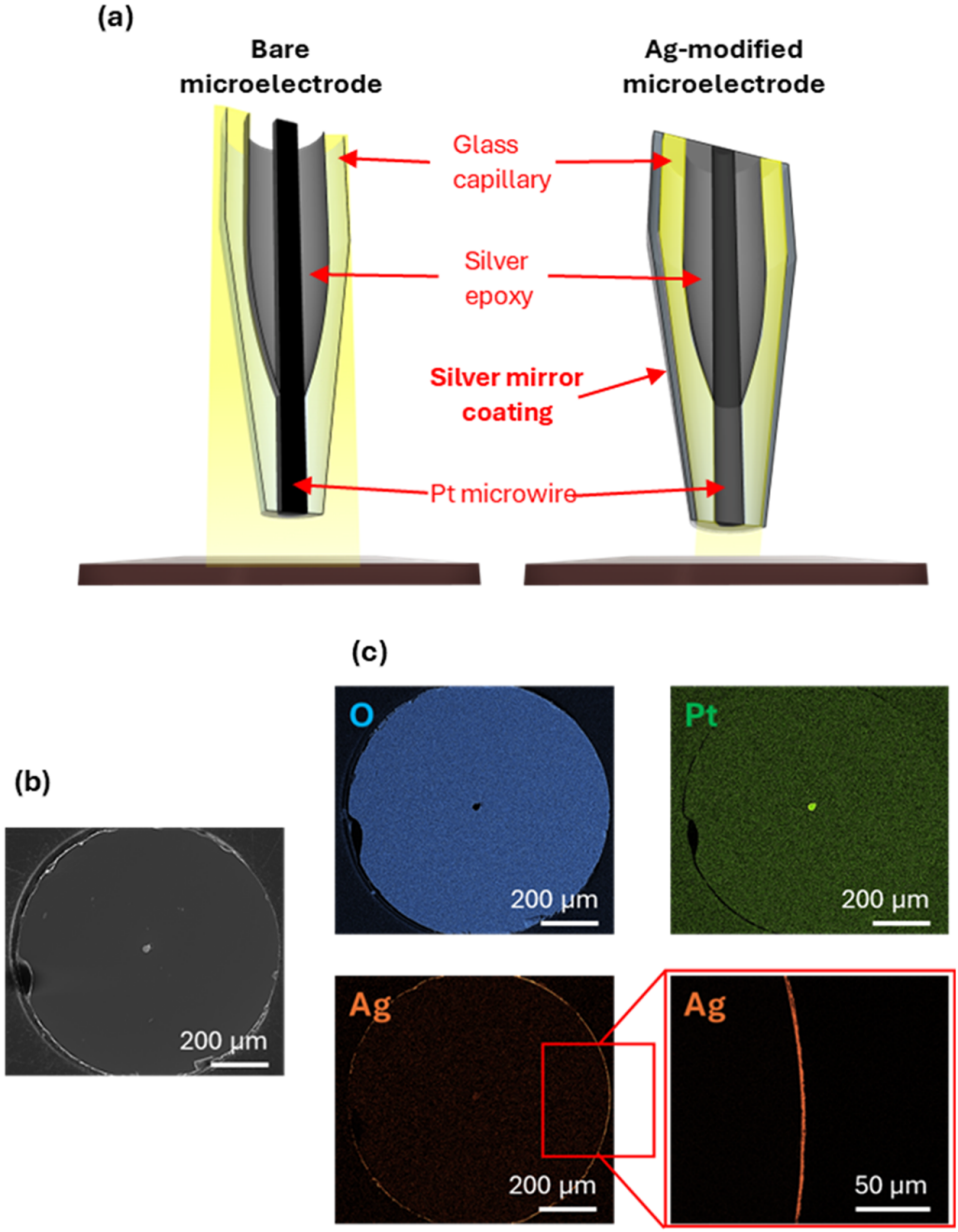
(a) Scheme of the illumination characteristic using a bare microelectrode and an Ag-covered microelectrode. (b) SEM images of an Ag-mirror-covered platinum-microelectrode and (c) the corresponding color-coded EDX elemental maps for oxygen, platinum, and silver.

Prior to the silver deposition, the glass sheath was first activated using SnCl_2_ in an acidic methanol solution to improve the surface adhesion of silver.^53^ They were then immersed in the freshly prepared Tollens’ reagent, to which glucose was added immediately prior to coating. Within several minutes of immersion, a uniform, bright silver mirror was formed on the outer wall of the glass capillary. After rinsing with ultrapure water, the electrodes were insulated with a transparent varnish to improve the stability of the coating and then gently repolished to restore a clean, exposed electroactive area surrounded by noncoated glass. The successful coverage of the glass sheath of the microelectrode with the Ag-coating was confirmed using scanning electron microscopy (SEM) and energy-dispersive X-ray spectroscopy (EDX), as shown in Figure 1b,c. The SEM image (Figure 1b) reveals a continuous and compact metallic layer surrounding the glass capillary, confirming the uniformity of the silver mirror. The corresponding EDX elemental maps (Figure 1c) clearly indicate the localization of Ag along the outer glass wall, while Pt and O signals are visible at the repolished end of the microelectrode.

Further, the light power was measured and revealed a significant enhancement of the local irradiance upon deposition of the reflective silver mirror. At a fixed tip–detector distance of 20 μm above the sample surface, for a noncoated microelectrode, a light intensity of 151  ±  3 W m^–2^ (*n* = 3) was measured, whereas the Ag-mirror-covered microelectrode exhibited a substantially higher intensity of 504 ±  10 W m^–2^ (*n* = 3), corresponding to a 3.3-fold increase in irradiance at the same working distance. To account for differences in the light beam spread and the total illuminated area, both microelectrodes (bare and covered) were approached toward a highly reflective substrate (gold) while being imaged by using a high-resolution optical camera. The resulting illumination characteristics (Figure S1) show a marked difference in beam confinement: the bare electrode produced a broad illumination area of 4.60 mm^2^, while the Ag-coated sensor generated a focused spot of only 0.15 mm^2^. This approximately 30-fold reduction in area, combined with the increased irradiance, reflects the improved spatial confinement and optical efficiency achieved by the silver mirror modification.

To quantify the illumination power at the sample in terms of photon output,^54^ the photon flux density (ϕ_p_) was calculated using eq 1:

1 $$\phi_{p}=\frac{I}{{N_{A}E}_{p}^{\lambda }}$$

where *I* is the experimental light intensity, *N*_A_ is the Avogadro number, and *E*_p_^λ^ is the photon energy at a specific wavelength. The photon energy was derived from the Planck–Einstein relation (eq 2), where *h* corresponds to the Planck constant (*h* = 6.62607015 × 10^–34^ J/Hz) and *c* is the speed of light in a vacuum (*c* = 29,97,92,458 m/s).

2 $$E_{p}^{\lambda }=\frac{\mathrm{hc}}{\lambda }$$

The calculations were performed at 470 nm, a wavelength in the visible blue region that is frequently used in photocatalytic studies owing to its partial overlap with the absorption bands of various semiconductors and molecular photocatalysts.^55^

Based on the experimentally measured irradiance and illumination areas, the corresponding molar photon flux densities were found to be 0.59 mmol photons /s m^2^ for the bare microelectrode and 1.98 mmol photons/ s m^2^ for the Ag-mirror-coated microelectrode. This yields an enhancement factor of approximately 3.4-fold, which transforms the glass capillary from a passive light waveguide into a confined reflective cavity that concentrates the photon flux toward the microelectrode apex.

Indeed, within the glass sheath of the noncoated microelectrode, the light beam exits through the transparent glass wall into air, where the refractive index contrast between glass (*n* = 1.5) and air (*n* = 1.0) generates only limited Fresnel reflection (approximately 4%) at each interface. Therefore, most of the light refracts outward at relatively large divergence angles, forming a broad and weakly collimated illumination cone. The energy density at the tip–sample junction is therefore small, and a significant fraction of photons is lost by refraction and scattering along the glass–air interface (Figure S1a).

For the Ag-coated microelectrode, the high reflectivity of silver (>95 % in the visible range) ensures that the photons incident on the inner wall of the glass sheath undergo efficient internal reflection rather than escaping through the glass–air interface. As a result, the Ag-coating behaves as an efficient reflective sheath, confining the light within the glass and effectively funneling the light toward the tip.

### 2.2 Preparation and Characterization of the Langmuir Films

As a proof of principle for the application of the Ag-covered microsensors in local photocatalysis, we selected molecular components that had already demonstrated efficient light-driven H_2_ evolution in biomimetic soft-matter environments. In a previous work, Abbas et al.^6^ showed that coembedding the amphiphilic bis­(2,2′-bipyridine)-(4,4′-dinonyl-2,2′-bipyridine)-ruthenium­(II) **RuC**_**9**_ PS and the HER CAT **[Mo**_**3**_ **S**_**13**_ **]** ^**2–**^ within liposomes showed promising photocatalytic activity in water in the presence of ascorbic acid (pH = 6) as a sacrificial electron donor. The zwitterionic phospholipidic bilayer provided an optimal microenvironment for charge separation and electron transfer between the excited PS and the CAT, owing to its gel–fluid phase coexistence near room temperature, which promotes molecular mobility while maintaining structural integrity.^6,56^

Building on these findings, we sought to translate the same PS–CAT combination from a closed bilayer (liposome) architecture^6^ into a planar, solid-supported lipid monolayer configuration to obtain a more durable and spatially controlled platform for photoactive assemblies with aligned building blocks.^24,57^ In contrast to liposomes, which are dispersed systems, Langmuir-type films provide a controllable 2D arrangement at the air–water interface, enabling systematic tuning of molecular packing through varying the lateral compression. Moreover, overall monolayer orientation can be controlled by applying either the vertical LB or horizontal LS deposition technique to transfer the film onto a solid substrate (Figure 2a). This approach bridges the gap between self-assembled soft-matter systems and surface-confined photocatalytic architectures, offering a pathway toward reproducible, transferable, and spatially organized photoactive layers that can be produced on a large scale.^58,59^

**Figure 2:**
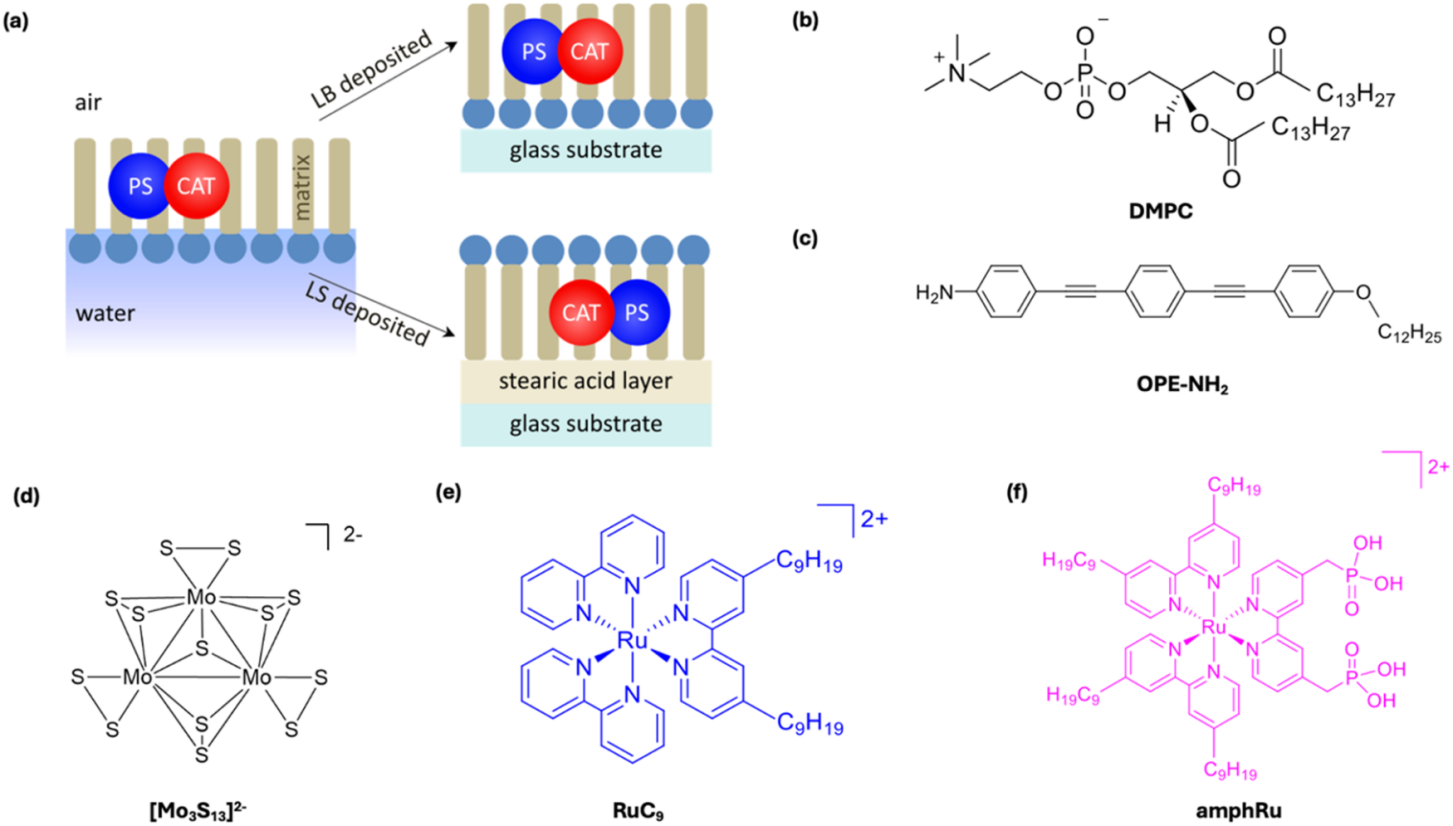
(a) Schematics of the Langmuir–Schaefer (LS) and Langmuir–Blodgett (LB) deposition configurations. In LS films, the hydrophobic alkyl chains of the matrix (e.g., **DMPC** or **OPE-NH**_**2**_) interact with a hydrophobized glass substrate via a **SA** monolayer, whereas in LB films the hydrophilic headgroups face the glass substrate, and therefore may expose the photocatalytic components toward the aqueous phase containing ascorbic acid (AA) as electron donor. (b) Molecular structure of the **DMPC** matrix. (c) Molecular structure of the **OPE-NH**_**2**_ matrix. (d) Molecular structure of the HER CAT **[Mo**_**3**_ **S**_**13**_ **]** ^**2–**^. (e) Molecular structure of the **RuC**_**9**_. (f) Molecular structure of the **amphRu**.

The dimyristoylphosphatidylcholine (**DMPC**) (Figure 2b) lipid was employed as a soft, zwitterionic matrix for the formation of the Langmuir films, while a more rigid π-conjugated matrix, oligo­(phenylene ethynylene) derivatives (**OPE–NH**_**2**_)^60,61^ (Figure 2c) containing n-dodecane substituents for correct alignment, was introduced to assess how matrix rigidity and intermolecular π–π interactions affect the packing and stability of the coembedded Ru-based PS and the **[Mo**_**3**_ **S**_**13**_ **]** ^**2–**^ CAT (Figure 2d). To probe the role of molecular anchoring, two Ru complexes were investigated: the **RuC**_**9**_ with hydrophobic moieties (Figure 2e), known to intercalate into hydrophobic environments, and the amphiphilic phosphonate-terminated Ru­(4,4′-dinonyl-2,2′-bipyridine)_2_(4,4′-bis­(phosphoric acid)-2,2′-bipyridine)^2+^ (**amphRu**) (Figure 2f), designed for a stronger interfacial interaction. Together, these systems enable a comparative study of how matrix composition and PS design influence supramolecular organization and the formation of stable, well-ordered photoactive films.

Using the two different deposition techniques (LB: vertical transfer of the monolayer onto the solid substrate and LS: horizontal transfer of the monolayer onto the solid substrate), as detailed below, allows a different molecular orientation and interfacial exposure of the catalytic domains, as illustrated in Figure 2a. The formation and organization of the Langmuir monolayers, pristine matrices, mixed films, and PS-only assemblies were monitored through surface pressure–mean molecular area Π­(mma) isotherms, from which the surface compressional modulus (*C*_s_^–1^) was derived (Figure 3a,b), together with Brewster angle microscopy (BAM) images acquired at 20 mN/m (Figure 3c–f). All isotherms show large linear regimes reaching to 38 mN/m and even higher surface pressures. This indicates the presence of single, well-defined, quasi-two-dimensional phases. The comparison of the spatial demand per molecule with molecular cross sections allows for a conclusion on the presence of monolayers. These spatial demands can be assessed using the mean molecular area at the maximum surface compression modulus (mma­(*C*_S,max_^–1^))­(Figure 3b), which corresponds to the steepest slope of the surface pressure-mean molecular area isotherm ((Figure 3a).

**Figure 3:**
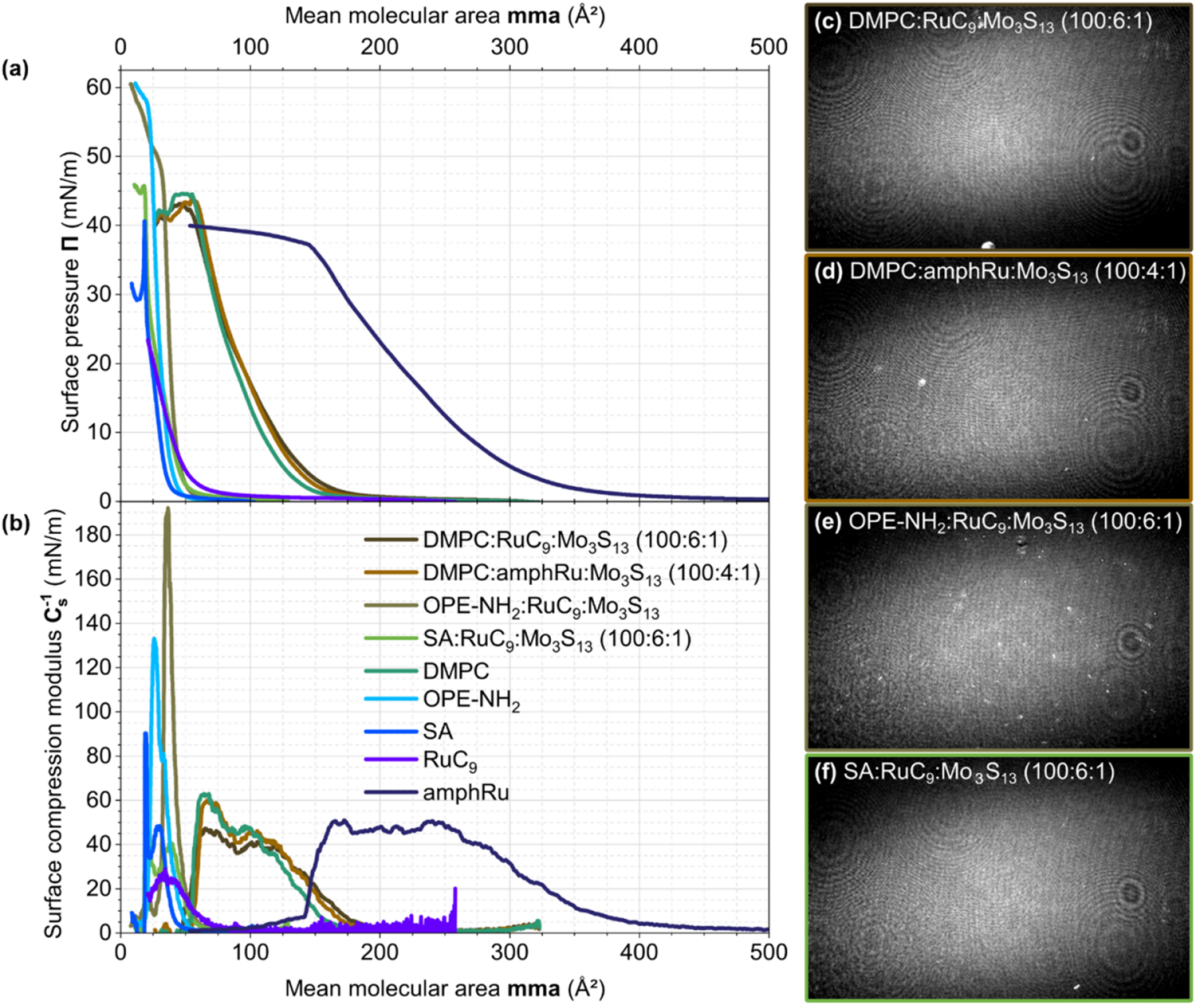
(a) Average surface pressure–mean molecular area (Π­(mma)) isotherms derived from three individual Π­(mma) isotherm measurements, respectively, and (b) corresponding surface compressional modulus (C_s_^–1^) plots of Langmuir films containing different Ru-based photosensitizers and matrices in the presence of **[Mo**_**3**_ **S**_**13**_ **]** ^**2–**^. (c–f) Brewster angle microscopy (BAM) images recorded at a surface pressure of 20 mN/m after 20 min stabilization, showing the morphology of the respective monolayers.

For **DMPC**, **SA**, and **OPE-NH**_**2**_, the mma-values at the maxima of the *C*/_S_^–^^1^ curves match the molecular cross sections, thus proving the presence of molecular monolayers. In the case of pristine **RuC**_**9**_, the small measured mma­(*C*_S,max_^–1^) = 42 Å^2^ demonstrates an exceptionally high packing density in the Langmuir layer.

For pristine **amphRu**, the extended steep linear isotherm segment gives rise to a broad plateau of the compressional modulus. The smallest mma in the plateau region is approximately 165 Å^2^, thus also matching the molecular cross-section and proving the presence of a monolayer. The corresponding molecular packing density in the monolayer (1/165 Å^2^ ≈ 0.6 molecules/nm^2^) is four times lower than that of the pristine **RuC**_**9**_ monolayer at its maximum compressional modulus (1/42 Å^2^ ≈ 2.4 molecules/nm^2^). **DMPC** shows maximum compressional moduli at 95 Å^2^ (liquid­(-crystalline) phase) and at 65 Å^2^ (solid phase). **OPE–NH**_**2**_ exhibits a maximum compressional modulus at a mean area of at 23 Å^2^, see (Figure 3b).

The small isotherm shifts to larger mmas for all mixed monolayers shown in Figure 3 indicate that PS and CAT components are incorporated into the matrices. Accordingly, the BAM images (Figure 3c–f) of the floating Langmuir layers recorded at a surface pressure of 20 mN/m show smooth and homogeneous Langmuir layers. However, in the case of the **DMPC**:**RuC**_**9**_:**[Mo**_**3**_ **S**_**13**_ **]** ^**2–**^ Langmuir layer, the isotherm reaches smaller mmas compared to that of pristine **DMPC** as soon as the surface pressure rises above 25 mN/m. Because the cationic center of **RuC**_**9**_ is shielded to a certain extent by the bipy ligands, we hypothesize that upon compression beyond 25 mN/m, **RuC**_**9**_ is gradually expelled from the monolayer. For the **DMPC** Langmuir monolayer containing small fractions of **amphRu** and **[Mo**_**3**_ **S**_**13**_ **]** ^**2–**^, the mean molecular area remains slightly larger than that of pristine **DMPC** even in the highly compressed state, indicating that **amphRu** is tightly anchored within the monolayer. Further studies of the Π­(mma) isotherms are presented in the Supporting Information (Section 2, Figures S2–S34). Overall, the interfacial data demonstrate that both DMPC and the corresponding OPE-NH_2_ matrices can accommodate small amounts of Ru-based PSs and **[Mo**_**3**_ **S**_**13**_ **]** ^**2–**^ clusters without notable distortion of the monolayer structure.

Total reflection X-ray Fluorescence (TXRF) measurements provided a direct elemental fingerprint of the **DMPC** and **OPE-NH**_**2**_ matrix LB and LS films, confirming the incorporation of the CAT. The presence of Ru originating from the PS was confirmed in all deposited films at the Ru Kα line (Figure S35a). All deposited films exhibit overlap of the Mo Lα and S Kα emission lines originating from the CAT (Figure S35b). Although these Mo/S contributions cannot be deconvoluted due to their intrinsic spectral overlap, the presence of a pronounced combined signal unambiguously confirms the incorporation of the Mo/S-based CAT into the films. Quantitative analysis further supports this conclusion; as summarized in Table S1, the mean count rates in the region of interest (ROI) at the Mo Lα line of all samples exceed the 3σ detection threshold determined from the blank measurement (LOD = 6.6 cps). This demonstrates that the detected Mo/S signals originate from the catalytic cluster rather than instrumental noise, verifying the presence of **[Mo**_**3**_ **S**_**13**_ **]** ^**2–**^ clusters in all of the investigated films.

X-ray photoelectron spectroscopy (XPS) analysis corroborates the incorporation of the Ru-based PS and the DMPC matrix (Figures S36 and S37). As shown in Figure S36, despite the partial overlap of the Ru 3d_5_/_2_ with the C 1s envelope, a distinct Ru contribution at a binding energy (BE) of ∼281.8 eV is detectable, consistent with Ru­(II) polypyridyl complexes, confirming the presence of **amphRu** even at very low loadings. The C 1s and N 1s regions further reflect the expected chemical signatures of the **DMPC** matrix and the bipyridyl ligand environment. The N 1s spectra (Figure S36e,f) show two chemically distinct environments: a component at a BE of ∼403.2 eV, assigned to the quaternary ammonium (N^+^) site of **DMPC**, and a component at a BE of ∼400.7 eV, attributed to the bipyridyl nitrogen atoms of the Ru-based PS. It should be noted that for a **DMPC** matrix:PS:CAT ratio of 100:4:1, the N^+^ signal originates from the **DMPC** matrix; the N_bpy_ peak originates from the Ru-based PS. XPS analysis has also been performed for the **OPE-NH**_**2**_ matrix, which is shown in Figures S38 and S39, respectively. Clear differences between the **DMPC** (N^+^ peak) signal and **OPE-NH**_**2**_ (NH_2_ peak) at a BE of ∼399.2 eV in the N 1s spectra are evident, indicating that we can detect both matrices. The PS is again visible with the small N_bpy_ peak at a BE of ∼400.7 eV and a distinct Ru 3d_5/2_ contribution that is observed at a BE of ∼281.8 eV, consistent with Ru­(II) in polypyridyl complexes. Further details and comparative spectra are provided in the Supporting Information (Section 4).

### 2.3 H_2_ Evolution Studies via SPECM

The Ag-coated microsensors were used in the SG/TC mode of SECM to locally measure the light-induced photocatalytic H_2_ evolution of the Langmuir films in 0.1 M ascorbic acid (pH = 4), as shown in Figure 4a, using a 470 nm LED fiber. The light was coupled directly into the Ag-coated glass sheath of the SECM microsensor to confine the illumination spot directly to the region of the H_2_ measurement. Under these conditions, we ensured that photocatalysis was activated exclusively within the illuminated footprint of the Langmuir film while the surrounding regions remained effectively in the dark.

**Figure 4:**
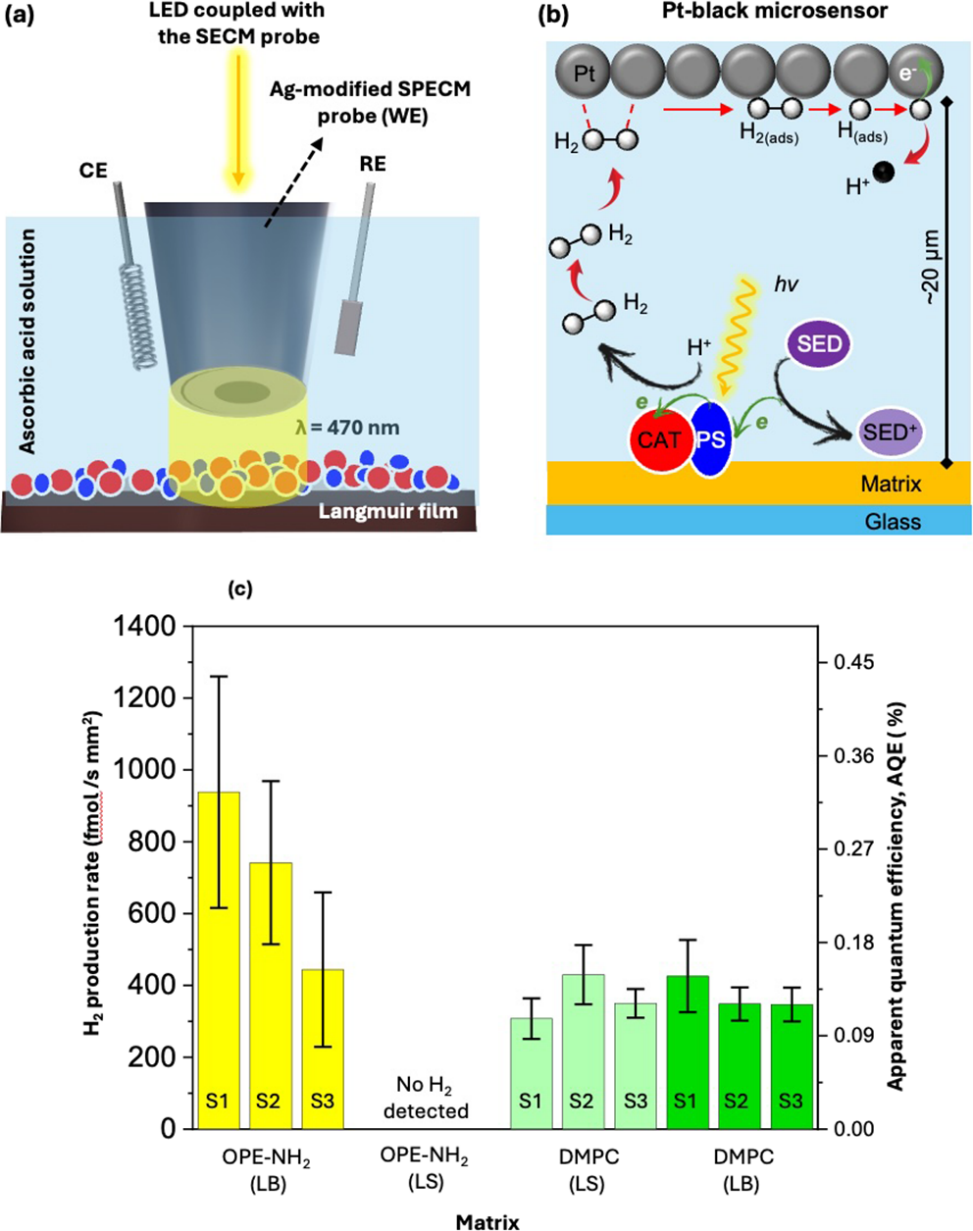
(a) Experimental setup employing the Ag-coated microsensor coupled to a 470 nm LED for spatially confined illumination of the photoactive Langmuir film immersed in 0.1 M ascorbic acid (pH = 4). (b) Schematics of the local photocatalytic process: The light excitation of the PS triggers electron transfer to the CAT, which reduces the number of protons to H_2_. The H_2_ diffuses (distance microsensor-to-surface: ∼20 μm) to the Pt-black probe, where it is oxidized back to protons, allowing quantitative detection. (c) H_2_ production rate and apparent quantum efficiencies (AQE) were determined from three spots on three independent samples (S1–S3) for the different film configurations. Yellow bars correspond to **OPE-NH**_**2**_-based LB films, while green bars represent **DMPC**-based LS (pale light green) and LB (light green) films coembedding **RuC**_**9**_ and **[Mo**_**3**_ **S**_**13**_ **]** ^**2–**^. The molar mixing ratio of matrix:**RuC**_**9**_:**[Mo**_**3**_ **S**_**13**_ **]** ^**2–**^ is 100:6:1. Error bars denote standard deviations over the measurements in three different regions of the same sample.

As schematically shown in Figure 4b, the photogenerated H_2_ at the Langmuir films diffuses toward the Pt-black-modified microsensor,^33^ positioned 20 μm above the modified Langmuir film. The H_2_ is adsorbed and oxidized at the electrode surface biased at −0.05 V vs Ag/AgCl. The corresponding charge is converted into molecular H_2_ production rates using Faraday’s law. These values were normalized by the illumination time and beam area, yielding local H_2_ evolution rates expressed in fmol/ s mm^2^. Figure S40 presents representative chronoamperometric curves recorded under photocatalytic (red trace) and dark (black trace) conditions using a LB film composed of **DMPC** as matrix, **RuC**_**9**_ as PS, and **[Mo**_**3**_ **S**_**13**_ **]** ^**2–**^ as CAT, immersed in a 0.1 M ascorbic acid solution (pH = 4) serving as a sacrificial electron donor (Figure S40a). Similar curves under illumination have been obtained for LS films with **DMPC** as matrix, **RuC**_**9**_ as PS, and **[Mo**_**3**_ **S**_**13**_ **]** ^**2–**^ as CAT; LB films **with DMPC** as matrix, **amphRu** as PS, and **[Mo**_**3**_ **S**_**13**_ **]** ^**2–**^ as CAT; and LB films with **OPE-NH**_**2**_ as matrix, **RuC**_**9**_ as PS, and **[Mo**_**3**_ **S**_**13**_ **]** ^**2–**^ as CAT (Figure S40b–d).

By controlling the illuminated area via local illumination and accurately quantifying the incident photon flux, the apparent quantum efficiency (AQE) of the photocatalytic process can be calculated. To assess how effectively the incident photons interacted with the PS, enabling the electron transfer to the CAT for H_2_ evolution, AQE was calculated for all films using (eq 3), which relates the number of evolved H_2_ molecules to the total number of incident photons.^62^ While H_2_ evolution rates quantify the absolute photoactivity, the AQE provides a more comprehensive performance metric by normalizing the product formation to the photon input, thus evaluating the photochemical conversion efficiency of each configuration.

3 $$\mathrm{AQE}\left(\%\right)=\frac{2\times \;\mathrm{number}\;\mathrm{of}\;\mathrm{evolved}\;H_{2}\;\mathrm{molecules}}{\mathrm{number}\;\mathrm{of}\;\mathrm{incident}\;\mathrm{photons}}$$

The number of incident photons was calculated based on the photon flux density (ϕ_p_), the illuminated area (*A*_beam_), and Avogadro’s number (*N*_A_), as shown in eq 4:

4 $$\mathrm{incident}\;\mathrm{photons}=\phi_{P}N_{A}A_{\mathrm{beam}}$$

Figure 4c summarizes the measured H_2_ production rates along with the AQE values for the four Langmuir monolayer configurations: LB-**OPE-NH**_**2**_, LS-**OPE-NH**_**2**_, LS-**DMPC**, and LB-**DMPC** using **RuC**_**9**_ as PS and **[Mo**_**3**_ **S**_**13**_ **]** ^**2–**^ as CAT. Each bar in Figure 4c represents measurements acquired from three different spots of the same sample, offering insight into the local variability and spatial heterogeneity within individual films. Groups of bars sharing the same color correspond to measurements taken on independently prepared samples (S1, S2, and S3), thereby reflecting the reproducibility of the fabrication and functionalization process across multiple preparations. These results reveal marked differences depending on both the matrix composition (**OPE-NH**_**2**_ or **DMPC**) and the deposition technique (LB or LS) used to determine the orientation of the matrix molecules on the glass substrate.

The LB-deposited **OPE-NH**_**2**_ Langmuir monolayers containing **RuC**_**9**_ and **[Mo**_**3**_ **S**_**13**_ **]** ^**2–**^ exhibited by far the highest photocatalytic activity, with H_2_ evolution rates approaching 1000 fmol/s mm^2^ and with quantum efficiencies of approximately 0.2–0.3%. However, they also showed the largest standard deviations. The strong variation among different spots within the same sample and between independently prepared samples indicates that, while highly active, the LB-deposited OPE-based assemblies might show large structural heterogeneity. In contrast, if the orientation of the Langmuir layers (i.e., **OPE-NH**_**2**_:**RuC**_**9**_:**[Mo**_**3**_ **S**_**13**_ **]** ^**2–**^) on the glass support is flipped through using horizontal LS (involves previous hydrophobization of the glass via a self-assembled SA layer) instead of LB deposition, no H_2_ was detected. This highlights the critical dependence of the activity on the molecular orientation and interfacial exposure of the catalytic domains.

The molecular arrangement and compatibility of PS and CAT within the matrix provide an explanation for these differences. The **RuC**_**9**_ complex, with the two long nonyl substituents, naturally tends to insert into hydrophobic environments. In flexible lipidic matrices such as **DMPC**, these alkyl tails interdigitate with the hydrocarbon chains of the phospholipidic matrix, while the positively charged Ru center remains near the polar interface, ensuring stable and well-oriented embedding. This furthermore ensures that the Ru center is easily accessible to ascorbate, the sacrificial electron donor, for the tunneling electron transfer between the Ru center and the e^–^ donor.

The **OPE-NH**_**2**_ matrix, however, consists of a rigid π-conjugated backbone that forms densely packed, planar domains.^60,61^ Such an aromatic network (π–π stacking) is poorly compatible with alkyl intercalation,^25,57,63^ likely causing partial segregation of **RuC**_**9**_ or the formation of small surface aggregates rather than a homogeneous comixed monolayer.^64^

This nanophase separation naturally leads to the pronounced heterogeneity and spot-dependent activity observed in LB-**OPE-NH**_**2**_ films. During LB deposition, the (glass) substrate moves upward through the monolayer, leading the hydrophilic headgroups—such as the terminal amines of OPE—to orient toward the glass substrate. If both **RuC**_**9**_ and the **[Mo**_**3**_ **S**_**13**_ **]** ^**2–**^ CAT reside mainly in the hydrophobic region of the film, then their active metal centers will thus face the solution. This conformation is advantageous for the photocatalytic process, as the photoactive sites remain exposed to the sacrificial electron donor in solution and are accessible to protons, enabling efficient light-driven H_2_ formation at the film–solution interface. In LS films, however, the situation is reversed: the hydrophobic chains attach to a SA-coated, hydrophobized substrate, leaving the hydrophilic moieties facing the external medium. Under these conditions, the **RuC**_**9**_ and **[Mo**_**3**_ **S**_**13**_ **]** ^**2–**^ units, embedded within the hydrophobic film, become partially or fully buried near the substrate. For **OPE-NH**_**2**_, the rigid aromatic backbone apparently separates the catalytic centers from the aqueous phase, preventing access of ascorbic acid and protons and thus completely suppressing the photocatalytic response. The contrasting performance between LB-**OPE-NH**_**2**_ and LS-**OPE-NH**_**2**_ films, therefore, may be directly associated with this direct geometric inversion of the molecular orientation, which determines whether the catalytic sites are exposed or rather encapsulated.

The **DMPC**-based films, in contrast, displayed moderate but rather uniform H_2_ generation regardless of the deposition mode, typically around 300 to 400 fmol/s mm^2^ (Figure 4c, green bars) and quantum efficiencies between 0.10 and 0.15%. The rather small standard deviations across different regions and samples point to the formation of highly uniform and reproducible films. The intrinsic amphiphilicity of the **DMPC**, with its dual hydrocarbon chains and zwitterionic phosphocholine headgroup, may enable stable monolayer formation that accommodates both **RuC**_**9**_ and **[Mo**_**3**_ **S**_**13**_ **]** ^**2–**^ within the film. The **RuC**_**9**_ PS likely intercalates into the lipid tails, as supported by the slightly larger mmas of **DMPC**:**RuC**_**9**_:**[Mo**_**3**_ **S**_**13**_ **]** ^**2–**^ vs pristine **DMPC** Langmuir monolayers at 20 mN/m, while the distribution of the CAT within the interfacial region is expected to depend on its interaction with both the matrix and the surrounding medium, influencing its accessibility under different deposition geometries. The overall lower activity of **DMPC** films compared to **OPE-NH**_**2**_ can be attributed to the insulating nature of the lipid matrix, which restricts long-range charge transport and electronic coupling between the embedded components. Nevertheless, the structural integrity and reproducibility of the **DMPC** layers make them significantly more robust under photocatalytic conditions.

To further assess the influence of the PS structure and its interaction with the matrix, additional experiments were performed using the same **DMPC**-based films but replacing the **RuC**_**9**_ complex with **amphRu**, which bears a more polar headgroup. Both complexes share a similar coordination environment around the Ru­(II) center but differ in their terminal substituents: While **RuC**_**9**_ carries two long alkyl chains that drive its partitioning into the hydrophobic regions, the **amphRu** introduces two phosphonic acid moieties that increase its affinity toward the polar and aqueous environments. Furthermore, **amphRu** can also deprotonate, which would also alter the overall charge of the PS up to 2^–^. This difference significantly alters the mode of incorporation within the **DMPC** film and, consequently, its interfacial orientation and photocatalytic behavior.

The results in Figure 5 reveal that the **DMPC**–**RuC**_**9**_ systems produce comparable H_2_ evolution rates for both LB and LS configurations, as previously shown. By contrast, the **DMPC** films containing the **amphRu** complex exhibited significantly lower H_2_ production rates and a pronounced sensitivity to the deposition geometry. In the LB configuration, a measurable photocatalytic response was observed (188 ± 17 fmol H_2_/s mm^2^), though reduced compared to **RuC**_**9**_, while in the LS configuration, no H_2_ evolution could be detected. This strong dependence on the layer architecture likely arises from the zwitterionic headgroup of the **amphRu** complex. The phosphonate moieties introduce a hydrophilic domain capable of hydrogen bonding and ionic interactions with the aqueous environment. At pH 4, which was used in all measurements, these phosphonic acid groups are expected to be fully protonated, reducing their anionic character and therefore avoiding electrostatic repulsion with the negatively charged CAT. This protonation may increase the mobility or desorption tendency of **amphRu** at the interface. In LB films, the phosphonate groups are oriented toward the solid substrate, while the hydrophobic nonyl chains extend away from the surface. This configuration may favor partial embedding of the **amphRu** complex within the **DMPC** matrix, maintaining some degree of spatial proximity to the **[Mo**_**3**_ **S**_**13**_ **]** ^**2–**^ CAT, allowing electron transfer. However, in the LS geometry, the amphiphilic structure likely inverts: The nonyl chains adhere to the hydrophobic substrate, while the polar phosphonate groups face outward. Given their protonated state at pH 4, these groups may no longer strongly coordinate or remain confined within the lipid–catalyst environment. Instead, they may promote partial solvation or desorption, likely decoupling the photoactive Ru centers from the catalyst layer and thereby possibly suppressing H_2_ evolution.

**Figure 5:**
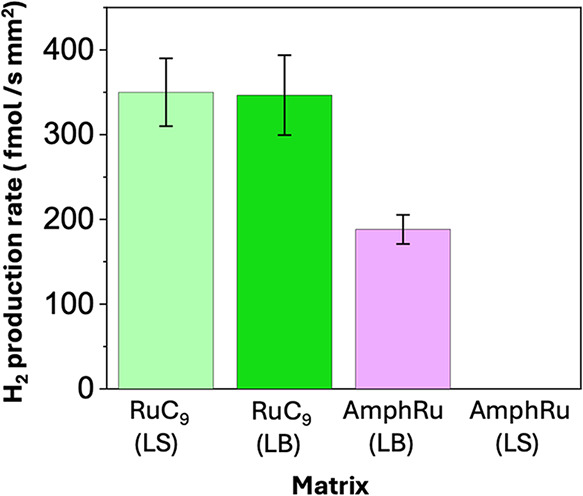
Local hydrogen evolution rates determined from chronoamperometric measurements under confined 470 nm illumination at **DMPC**-based-LS films using **RuC**_**9**_ as PS (pale green), at **DMPC**-based-LB films using **RuC**_**9**_ as PS (green), and **DMPC**-based-LB films using **amphRu** as PS (violet). **DMPC**-based-LS films using **amphRu** as the PS do not show H_2_ evolution over time. The molar mixing ratio of **DMPC:RuC9:[Mo**_**3**_ **S**_**13**_ **]** ^**2–**^ is 100:6:1, and that of **DMPC**:**RuC9**:**[Mo**_**3**_ **S**_**13**_ **]** ^**2–**^ is 100:4:1. Error bars represent standard deviations of measurements on three independent spots per film.

## 3 Conclusion

This study presents Ag-mirror-coated bifunctional microsensors that enable colocalized illumination and H_2_ measurements at spots on soft photocatalytic interfaces. The reflective Ag-coated sheath, deposited via the Tollens reaction, efficiently confines and directs light (470 nm) toward the microsensor apex, increasing the photon flux density by approximately 3.4-fold and reducing the illuminated area by nearly 2 orders of magnitude compared to a noncoated microelectrode. This optical confinement allows for quantitative correlation between local light input and product generation within micrometric regions of the photoactive films.

The Ag-coated H_2_ microsensors were used to determine the light-induced H_2_ evolution at different photoactive Langmuir films (modified with a Ru-based PS and **[Mo**_**3**_ **S**_**13**_ **]** ^**2–**^ as the HER CAT). The measurement revealed a direct structure–function relationship among molecular orientation, matrix rigidity, and photocatalytic performance. **OPE-NH**_**2**_–based films displayed the highest H_2_ evolution rates (ca. 1000 fmol/s mm^2^). However, these layers show spatially inhomogeneous and highly layer-orientation-dependent catalytic performance. In contrast, the **DMPC**-based films yielded lower but reproducible H_2_ evolution rates (ca. 300–400 fmol/s mm^2^) with a stable apparent quantum efficiency around 0.1–0.15%. The contrast between LB and LS geometries demonstrates that even a subtle inversion of the interfacial polarity of the matrix can determine whether photocatalytic activity is retained or suppressed, depending on whether the photoactive domains are exposed to the interface or buried within the matrix. A clear correlation between the nature of the PS and the overall photocatalytic activity could be shown, where lipophilic overall nonpolar complexes resulted in higher catalytic activity than lipophobically substituted complexes.

These findings highlight the advantages of confined photoelectrochemical microscopy to deconvolute structural and functional heterogeneities in photocatalytic systems. Beyond the present proof of concept, the Ag-coated microelectrodes open a route to the quantitative mapping of photon-to-fuel conversion efficiencies, possible heterogeneities, and to the rational design of robust, orientation-controlled hybrid films for artificial photosynthesis.

## 4 Experimental Section

### 4.1 Chemicals

Ammonia (NH_3_), acetone, L­(+) ascorbic acid, methanol, nitric acid (HNO_3_), silver nitrate (AgNO_3_), and sodium hydroxide (NaOH) were purchased from VWR (Leuven, Belgium). Chloroplatinic acid (H_2_PtCl_6_), glucose monohydrate (C_6_H_12_O_6_ H_2_O), hydrochloric acid (HCl), lead nitrate ((PbNO_3_)_2_), potassium chloride (KCl), phosphate-buffered saline (PBS), and tin chloride (SnCl_2_) were purchased from Merck Co. (Darmstadt, Germany). All solutions were prepared with ultrapure water (Elga system, UK, conductivity 18.0 MΩcm). Glass capillaries were obtained from Hilgenberg (Malsfeld, Germany), and platinum microwires were obtained from Goodfellow Cambridge Limited (Huntington, England). Polishing supplies (diamond lapping films and polishing suspensions) were purchased from Allied High-Tech Products, Rancho Dominguez (USA).

### 4.2 Fabrication of Microelectrodes for SPECM Studies

Microelectrodes were prepared following a well-established procedure described elsewhere.^65^ Briefly, a 25 μm diameter Pt-wire was sealed under vacuum in a pulled borosilicate capillary, followed by polishing on diamond lapping films (size 9 to 0.1 μm) with water and on cloth with a polishing suspension (particle size 0.05 μm) until a smooth surface was achieved. The microelectrodes were rinsed with ultrapure water and characterized by using optical microscopy and cyclic voltammetry.

To apply the silver mirror-like coating, the Tollens’ reagent^50^ was used as follows: prior to the coating, the glass sheath of the microelectrode was cleaned with acetone. The microelectrode was first activated by immersion in a sensitizing solution containing SnCl_2_ (1 g) dissolved in 8 mL of methanol with one drop of concentrated HNO_3_ for 2 min, followed by thorough rinsing with ultrapure water. The Tollens reagent was prepared by adding aqueous ammonia dropwise to 30 mL of 0.1 M AgNO_3_ until the initially formed brown precipitate dissolved, yielding the diamminesilver­(I) complex. Subsequently, 6 mL of 0.1 M NaOH was added to reform the silver oxide precipitate, which was again dissolved by careful addition of ammonia. Finally, 10 mL of a 1 M glucose solution was added to 30 mL of the freshly prepared Tollens’ reagent to initiate the reduction of Ag^+^ to metallic silver. The microelectrode was immediately immersed in the reaction mixture for 10 min to allow silver deposition and then subsequently rinsed thoroughly with ultrapure water. A layer of insulating varnish (RS components, Corby, Northants) was applied to protect the silver mirror coating. The electrode was again polished following the previously described procedure to remove any depositions from the electroactive area of the disc-microelectrode.

The electrochemical deposition of Pt-black was performed as described elsewhere^33,66^ using a three-electrode setup with the coated microelectrodes as the working electrode, an Ag/AgCl, KCl (3M) reference electrode, and a platinum wire serving as the counter electrode. A CHI660C potentiostat (CH Instruments, Austin, Texas, USA) was used to control the electrochemically induced deposition of Pt-black. For the electrodeposition, the microelectrode was immersed in a PBS solution containing 31 mM H_2_PtCl_6_ and 0.67 mM Pb­(NO_3_)_2_, applying a constant potential of −0.06 mV vs Ag/AgCl, KCl (3M) for 40 s.

### 4.3 Embedding and SEM/EDX Mapping

The Ag-coated 25 μm Pt microelectrode was placed vertically in a cylindrical mold. The mold was filled with EpoFix (Struers GmbH, Germany) until the tip of the Ag-coated microelectrode was completely immersed. Two vacuum steps (1 min, 230 mbar) were applied, followed by hardening for 12 h at room temperature. Afterward, the nonembedded part of the Ag-coated microelectrode was removed to achieve a flat surface by cutting and polishing with SiC abrasive paper (Struers GmbH, Germany). To expose the embedded tip of the microelectrode, SiC abrasive paper (8 μm, Leco Corporation, USA) and diamond particle suspensions (6, 3, and 1 μm, Leco Corporation, USA) on woven satin cloth (Gold Technotron, Leco Corporation, USA) were used using a grinding and polishing machine (PX400, Leco Corporation, USA).

SEM and EDX mappings were done on a Quanta FEG 3D (Thermo Fisher Scientific, USA) equipped with an Element EDX detector using the APEX standard software package (both EDAX Inc., Germany). SEM images were recorded by using an acceleration voltage of 5 keV and a beam current of 53.3 pA. EDX mappings were performed at 10 keV and 8 nA using a dwell time of 50 μs per pixel and 32 frames in total.

### 4.4 Local Light Intensity Measurements

The light intensities achieved with the Ag-coated and bare microelectrodes were measured by positioning them at a distance of 20 μm (typically used for the in situ localized activity measurements) above a power and energy meter used in combination with optical power monitor software (both Thorlabs GmbH, Germany). The microelectrodes were attached to a 1000 μm light fiber (Thorlabs GmbH, Germany) and coupled to a λ = 470 nm LED (Thorlabs GmbH, Germany). The power was controlled by an LED Driver (T-Cube, Thorlabs GmbH, Germany). The LED Driver was set to the highest mode during the measurements.

### 4.5 Hydrogen Evolution Studies via SPECM

All photocatalytic experiments were performed using a SECM instrument from Sensolytics GmbH & Co. KG (Bochum, Germany) in combination with an Autolab bipotentiostat (Metrohm Deutschland GmbH, Filderstadt, Germany). A three-electrode setup was used with the modified Pt–B microsensor as the working electrode, an Ag/AgCl pseudoreference electrode, and a platinum counter electrode. A 470 nm LED fiber (Thorlabs GmbH, Bergkirchen, Germany) was mounted in a custom holder that positioned the fiber tip coaxially above the microelectrode, ensuring stable vertical alignment. In this geometry, the glass capillary surrounding the electrode acted as a waveguide, directing the incident light toward the apex for localized illumination of the Langmuir films. The microsensor was then positioned over the Langmuir films immersed in 0.1 M ascorbic acid (pH = 4). To position the Pt-black microsensor, the oxygen reduction reaction at an applied potential of −0.6 V vs Ag/AgCl was used. After positioning, the solution was exchanged with a degassed (purged for 15 min with Ar) 0.1 M ascorbic acid. The photocatalytic experiments were then performed in 0.1 M ascorbic acid, at a probe potential of −0.05 V vs Ag/AgCl and a probe–surface distance of approximately 20 μm.

### 4.6 Langmuir Films Preparation

For Langmuir, Langmuir–Blodgett (LB), and Langmuir–Schaefer (LS) film preparation, dichloromethane (Carl Roth, ROTISOLV, min. 99,8%, UV/IR-Grade) solutions of 1,2-dimyristoyl-*sn*-glycero-3-phosphocholine (**DMPC**; matrix (M)), bis­(2,2′-bipyridine)-(4,4′-dinonyl-2,2′-bipyridine)-ruthenium­(II) (**RuC**_**9**_; PS), the phosphonate-terminated Ru­(4,4′-dinonyl-2,2′-bipyridine)_2_(4,4′-bis­(phosphoric acid)-2,2′-bipyridine)^2+^ (**amphRu**; PS), and the molybdenum sulfide cluster (NH_4_)_2_[Mo_3_S_13_] (CAT) were prepared at a concentration of 0.1 μmol/mL. The synthesis of **RuC**_**9**_ ^67^ and (NH_4_)_2_[**Mo**_**3**_ **S**_**13**_][Bibr ref13] has been previously reported in the literature. For synthesis details, the reader is directed to these publications. **amphRu** was synthesized from the silyl-ester-protected phosphonic acid according to a modified deprotection procedure by Amthor et al., and isolated as the hexafluorophosphate salt.^68^ The results of the characterization of **amphRu** agreed with the data available in the literature for this complex.^69^ The solutions were mixed to obtain a M:PS:CAT molar mixing ratio of 100:6:1 for **RuC**_**9**_-containing solutions and 100:4:1 for **amphRu-containing** solutions and a final concentration of 0.1 μmol/mL, respectively.

### 4.7 Characterization of the Langmuir Films

To record surface pressure vs mean molecular area (Π­(mma)) isotherms, 200 to 1000 μL of the M:PS:CAT solution was spread onto ultrapure water (0.16–0.25 μS/cm) in a temperature-controlled LB trough (KSV 5000; trough length 520 mm, compression length 475.2 mm, width 150 mm) maintained at 25 °C. After spreading, the films were allowed to equilibrate for 20 min to ensure complete solvent evaporation. Compression was then initiated at a maximum barrier speed of 10 mm/min, with an additional rate limitation of 5 mN/m min applied in the event of a rising surface pressure; otherwise, the maximum compression rate was used. For each composition, three to four isotherms were acquired, averaged, and plotted with corresponding error bands. The surface compression modulus *C*_s_^1–^ was calculated from the averaged Π­(mma) isotherm by applying eq 5:

5 $$C_{S}^{-1}=-mma\cdot \left(\frac{d\Pi }{dmma}\right)$$

For Brewster angle microscopy (BAM), a KSV NIMA MicroBAM 3 (Biolin Scientific) mounted on a KSV 5000 LB trough was used. BAM images were acquired before and after spreading, as well as continuously during film compression and at defined surface pressures. Spreading volumes between 900 and 2300 μL were employed. For LB and LS film preparation, a KSV NIMA KN 2006 trough (length of 746 mm, compression length of 560 mm, width of 75 mm) was used. Spreading volumes of 600–1700 μL were deposited onto the ultrapure water subphase, and the monolayers were compressed to a target surface pressure of 20 mN/m. At this pressure, the Langmuir film was equilibrated and subsequently transferred onto hydrophilic substrates (glass or silicon) by upward LB deposition at 1 mm min^–1^, allowing simultaneous coating of three to four substrates. After the LB transfer, three manual LS depositions were performed at different positions of the same Langmuir film onto hydrophobic substrates. Hydrophobic surfaces were prepared by forming a self-assembled monolayer (SAM) (5 mM SA in ethanol, Rotisolv HPLC grade, ≥99.9%, Carl Roth) on glass or by using isopropanol-stored hydrophobic silicon wafers.

To confirm the incorporation of all components within the Langmuir films, total reflection X-ray fluorescence (TXRF) and X-ray photoelectron spectroscopy (XPS) analyses were performed. TXRF measurements were performed using a high-efficiency module S2 Picofox (Bruker Nano GmbH, Berlin, Germany) equipped with a molybdenum X-ray tube. The samples were excited under maximum power conditions (50 kV and 600 μA) and measured using a live time of 1000 s. **DMPC**-based LS and LB samples were deposited on precleaned, nonsiliconized quartz glass sample carriers. To ensure film integrity and to prevent damage caused by the TXRF measuring cassette, a self-designed adhesive film (Silver, Cricut, Inc., South Jordan, UT, USA) cut using a plotter with a circular cutout (1 cm diameter) in the middle, was attached onto the sample carrier prior to sample preparation. The adhesive film was removed before the TXRF measurement. All prepared sample carriers, as well as a sample carrier without a sample, were measured nine times in total with a rotation of 45° after each measurement.

XPS was conducted using a K-α spectrometer (ThermoScientific) with a monochromatic X-ray source (Al K_α_) with a spot diameter of 400 μm and an electron detector with a 0.5 eV energy resolution. An internal flood gun was employed for charge compensation. The spectra were calibrated using the Si 2p peak (103.5 eV) and fitted using Voigt functions after background subtraction. As the Ru 3d_3/2_ peak is covered by the C 1s species, the Ru 3d doublet was fitted with a binding energy distance between Ru 3d_5/2_ and 3d_3/2_ of 4.2 eV, an intensity ratio of 3:2, and the same fwhm.

## Supplementary Material



## Data Availability

Data will be made available on request.

## Notes

Characterization of the Langmuir film, including Π­(mma) isotherms, BAM, TXFR, XPS, and chronoamperometry (PDF)
