# Supplementary material for: Scanning Photoelectrochemical Microscopy for the Investigation of Local Photocatalytic H2 Evolution in Matrixed Langmuir Films
Source: ACS Appl Mater Interfaces. 2026 May 1;18(18):26144–56. doi: 10.1021/acsami.5c26195 (PMC13181728; doi:10.1021/acsami.5c26195)
Supplement: Supplementary file 1 [file am5c26195_si_001.pdf]

Supporting Information

Scanning photoelectrochemical microscopy for the investigation of local photocatalytic H_2 Evolution in matrixed Langmuir films

Sarah Horn[a], Giada Caniglia[a], Sarah Jasmin Finkelmeyer[b], Gregor Neusser[a], Charlotte Mankel[c], Benedikt Bagemihl[d], Sven Rau[d], Christof Neumann[e,f,h], Andrey Turchanin[e,f,h], Riccarda Müller[a], Kerstin Leopold[a], Sidra Akther[g], Andrea Pannwitz[f,g,h], Stefan Zechel[c,h,i], Martin D. Hager[c,f,h,i,j,], Ulrich S. Schubert[c,f,h,i,j,l], Moritz Jahn[k], Carsten Streb[f,k], Martin Presselt[b,f,l]*, Christine Kranz[a]*

[a] Institute of Analytical and Bioanalytical Chemistry, Ulm University, Albert-Einstein-Allee 11, 89081 Ulm, Germany

[b] Leibniz Institute of Photonic Technology (IPHT), Albert-Einstein-Str. 9, 07745 Jena, Germany

[c] Laboratory of Organic and Macromolecular Chemistry (IOMC), Friedrich Schiller University Jena, Humboldtstr. 10, 07743 Jena, Germany

[d] Institute of Inorganic Chemistry I, Ulm University, Albert-Einstein-Allee 11, 89081 Ulm, Germany

[e] Institute of Physical Chemistry and Abbe Center of Photonics, Friedrich Schiller University Jena, Lessingstrasse 10, 07743 Jena, Germany

[f] Center for Energy and Environmental Chemistry Jena (CEEC Jena), Friedrich Schiller University Jena, Philosophenweg 7a, 07743 Jena, Germany

[g] Institute for Inorganic and Analytical Chemistry (IAAC), Friedrich Schiller University Jena, Humboldtstraße 8, 07743 Jena, Germany

[h] Jena Center for Soft Matter (JCSM), Friedrich-Schiller-University Jena, Philosophenweg 7, 07743 Jena, Germany

[i] Helmholtz Institute for Polymers in Energy Applications Jena (HIPOLE Jena), Lessingstraße 12–14, 07743 Jena, Germany

[j] Helmholtz-Zentrum Berlin für Materialien und Energie, Hahn-Meitner-Platz 1, 14109 Berlin, Germany

[k] Department of Chemistry, Johannes Gutenberg University Mainz, Duesbergweg 10-14, 55128 Mainz, Germany

[l] sciclus GmbH & Co. KG, Moritz-von-Rohr-Str. 1a, 07745 Jena, Germany

* Corresponding authors. Email: martin.presselt@leibniz-ipht.de, christine.kranz@uni-ulm.de

1. Optical images of illumination spots through the microelectrode sheaths

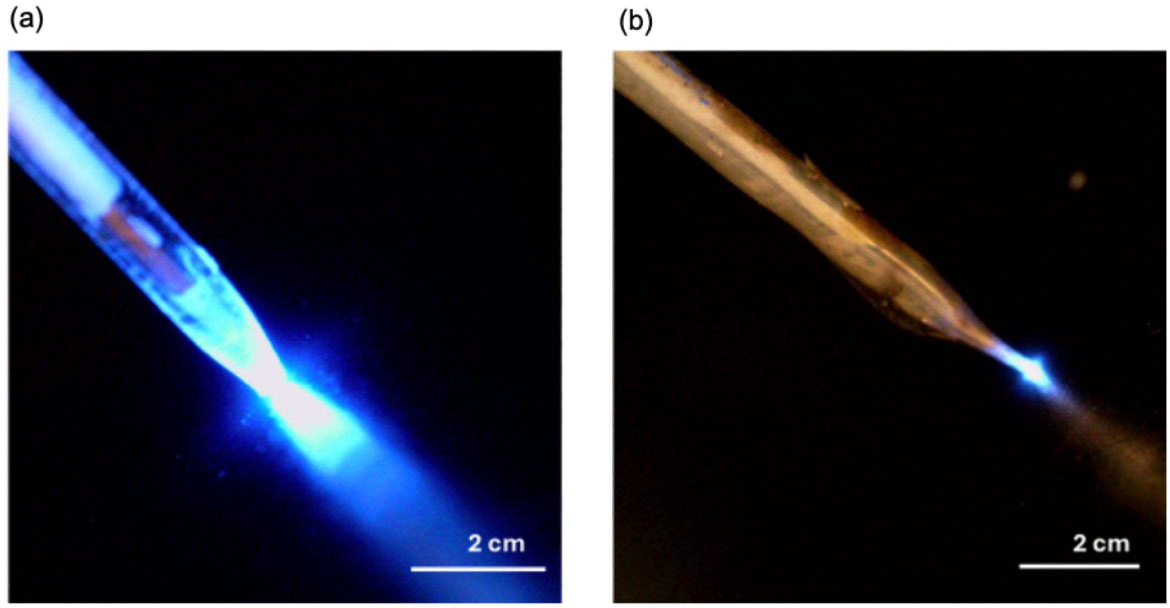

Figure S1. (a) Optical photograph of the bare SECM microelectrodes coupled with a 470 nm LED, captured using a digital camera. (b) Optical photograph of the silver-covered SECM microelectrodes coupled with a 470 nm LED, captured using a digital camera.

2. Π(mma) isotherms and Brewster angle microscopy (BAM)

2.1. Π(mma) isotherm analysis

For Π(mma) isotherm data analysis the JuPytR notebooks[4, 5] were utilized, namely using Python3[6] within Anaconda environment. The program takes raw data from Π(mma) isotherm measurements, processes each file separately, and then averages the data and uncertainties for each experiment's Π(mma) isotherms.

The surface compression modulus C_S^{-1} is calculated using the analytical first derivative of the averaged interpolation functions of the experimentally obtained Π(mma) isotherms:[7]

$$C_S^{-1} = -mma \cdot \left(\frac{\mathrm{d}\Pi}{\mathrm{d}mma}\right) \qquad (1)$$

Gaussian function is taken as the sum of Gaussian peak with an additional linear function:

$$f(x) = m \cdot (x - x_c) + \frac{A}{w\sqrt{\frac{\pi}{2}}} \exp\left(-\frac{x - x_c}{w}\right)^2 \qquad (2)$$

The function takes an input x and parameters: m, which is the linear coefficient that scales the linear component of the model. x_c is the x-coordinate of the centre of the Gaussian peak. A is the amplitude of the Gaussian peak, determining its magnitude. w is the standard deviation of the Gaussian peak, controlling the spread of the peak.

2.2. Algorithm of the program

The input file specifies boundaries for fitting parameters, the fitting range, initial guesses, and points of interest. The data within this range is fitted using a Gaussian function, with initial guesses and boundary constraints from the input file.

Optimization is done using the Levenberg-Marquardt algorithm to minimize the differences between model predictions and observed data points.[9-15] Standard deviations are calculated using the same optimization function. After fitting, the function returns optimal parameter values and a covariance matrix,[16] which shows the estimated covariance between fitted parameters. The variances of the fitted parameters are found on the diagonal of the covariance matrix.

The uncertainty of mma_C is taken as approximately equal to the uncertainty of x_c. The position of mma_C is determined by maximum of the fitting function. Therefore:[8]

$$mma_C Err = \sqrt{xcErr^2 + mma_C meanErr^2} \qquad (3)$$

But as no mean error is presented for a single Π(mma) isotherm, then one takes:

$$mma_C Err \approx xcErr. \qquad (4)$$

The corresponding surface pressure Π_C is also reported. The program then performs linear fitting within a specified range on the Π(mma) isotherm part of the subplot and calculates corresponding uncertainties. For linear extrapolation of the Π(mma) isotherm narrow data ranges whose mean molecular areas correspond to the top of the compression modulus peaks are used. The fitting range for linear fit is taken as w/3 around the position of the maximum point mma_c to obtain extrapolated mean molecular areas mma_0.

2.3. Average Π(mma) isotherm analysis

Since the mean molecular area points may vary between different Π(mma) isotherms, the data files are first extrapolated to cover the range from the minimum possible mean molecular area to the maximum among the isotherms being averaged. A uniform x-axis is introduced for this extrapolation. After extrapolation, the surface pressure values are averaged across different data files. The resulting average Π(mma) isotherm is then used for all subsequent procedures described earlier. The program saves the resulting columns into a .csv file, including mean molecular area, average surface pressure, mean molecular area error, compression modulus, and Gaussian function.

To calculate averages for specific points of the isotherms, the values are extracted from the outputs of the individual Π(mma) isotherms. These values are then averaged, and for uncertainties, a specific formula is applied:

$$\Delta\bar{x} = \frac{\sigma}{\sqrt{n}} \qquad (5)$$

Where n is the number of different files (isotherms), for which the specific value was calculated and σ is the standard deviation. Then for each of the values on average isotherm one has the value:

$$\bar{x} \pm \Delta\bar{x} \qquad (6)$$

Where $\bar{x}$ is the average of the value. This is done for mma_c, $C_{s,max}^{-1}$, Π_C and mma_0, which are, correspondingly, mean molecular area of collapse point, maximum of compression modulus, pressure of the collapse point and the point of intersection of the interpolated linear approximation of the isotherm with the mean molecular area axis.

2.4. Detailed and systematic study of individual and averaged Π(mma) isotherm and in-situ BAM recording

2.4.1. Averaged surface pressure (mean molecular area) (Π(mma)) isotherms and summary of BAM monitoring

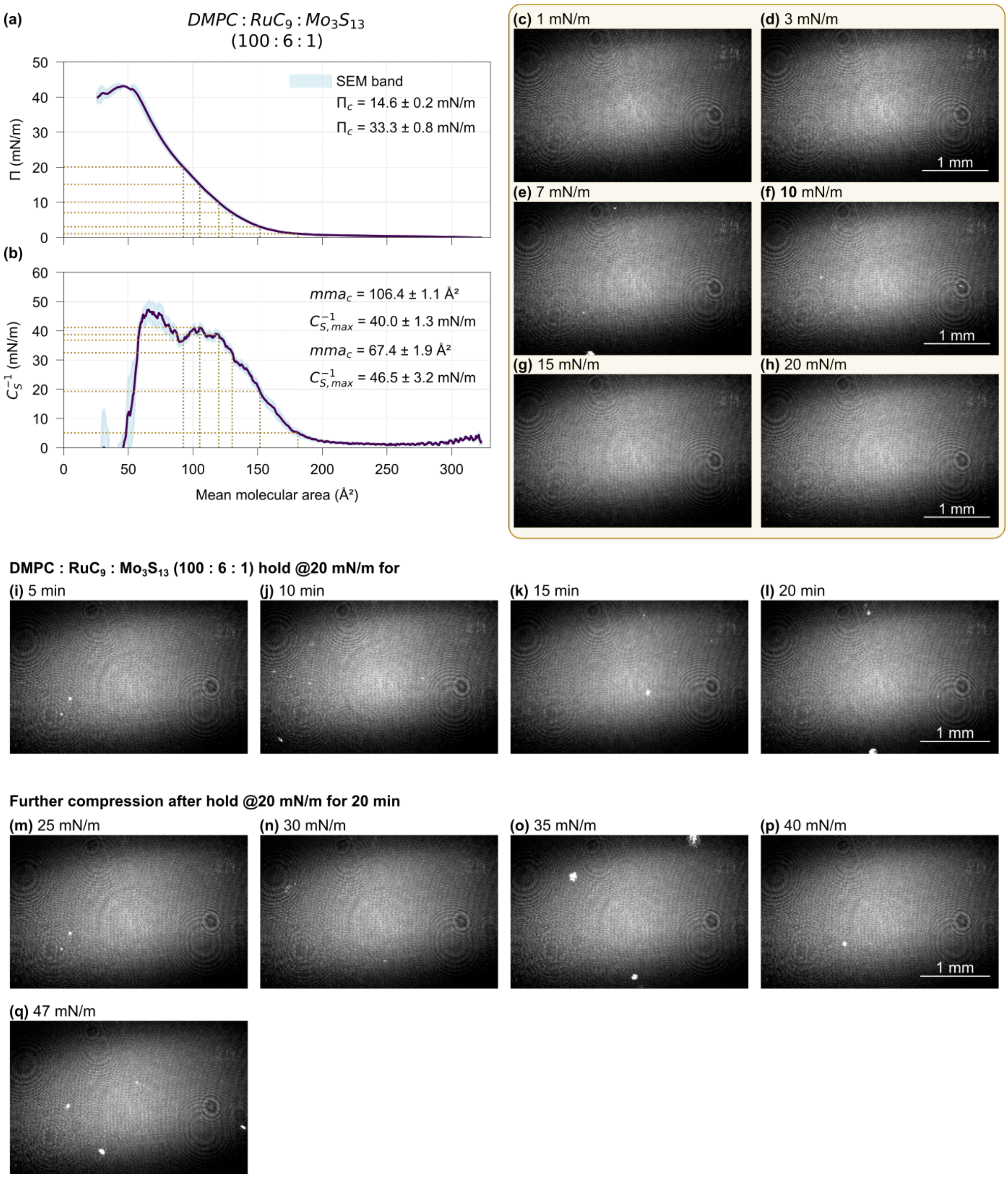

Figure S2: Π(mma) isotherm analysis of **DMPC**:**RuC$_9$:[Mo$_3$S$_{13}$]$^{2-}$** mixture with molar mixing ratio [%] **100:6:1** in (a) and (b). Curves represent the mean of three individual Π(mma) isotherm recordings with the standard error of the mean (SEM) indicated as a band. (c) to (h) BAM images of the course of the Π(mma) isotherm up to 20 mN/m. (i) to (l) hold at 20 mN/m for different time duration. (m) to (t) BAM images of further compression of the Langmuir film after duration of hold at 20 mN/m.

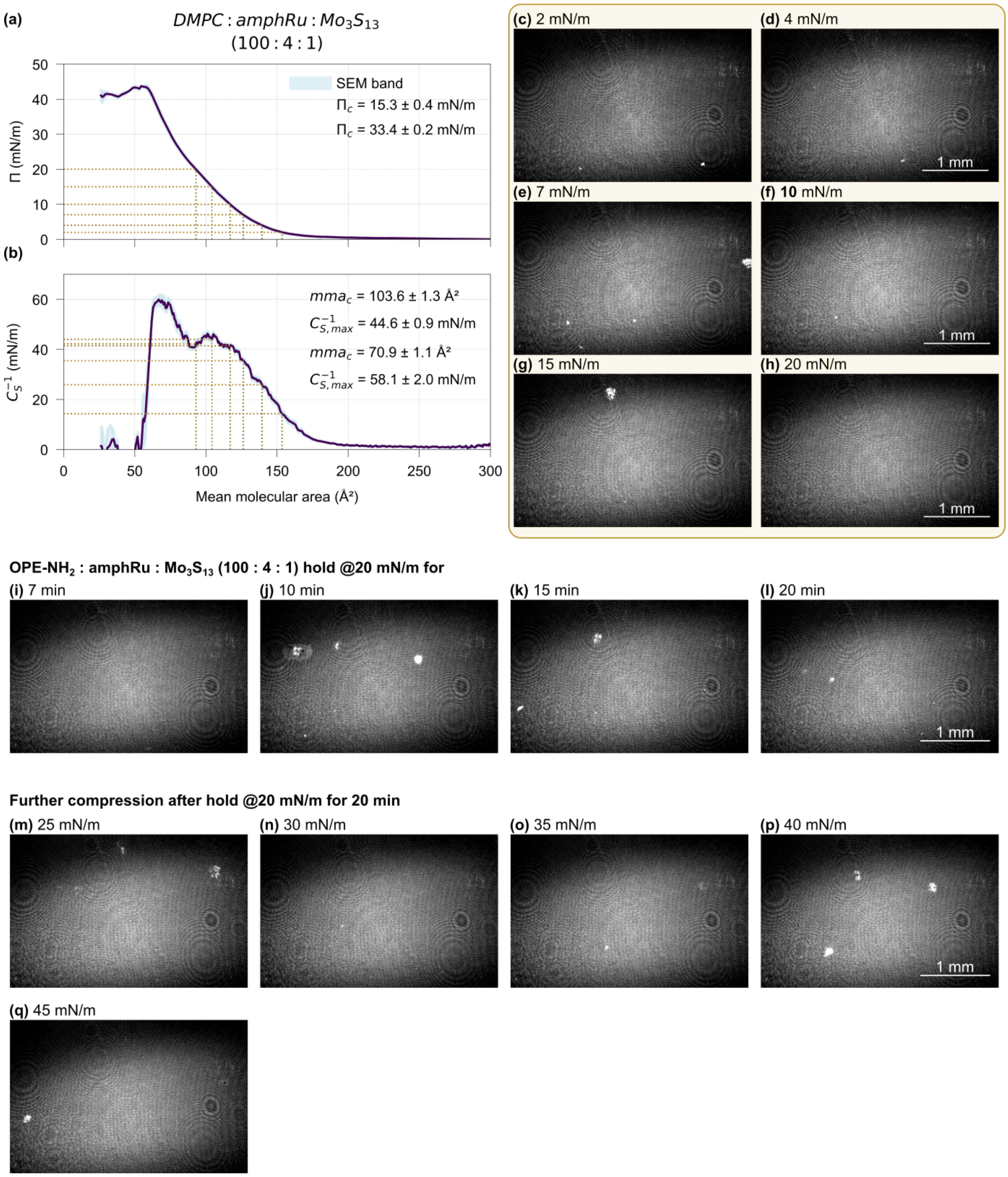

Figure S3: Π(mma) isotherm analysis of **DMPC**:**amphRu:**$\mathbf{[Mo_3S_{13}]^{2-}}$ mixture with molar mixing ratio [%] **100:4:1** in (a) and (b). Curves represent the mean of three individual Π(mma) isotherm recordings with the standard error of the mean (SEM) indicated as a band. (c) to (h) BAM images of the course of the Π(mma) isotherm up to 20 mN/m. (i) to (l) hold at 20 mN/m for different time duration. (m) to (t) BAM images of further compression of the Langmuir film after duration of hold at 20 mN/m.

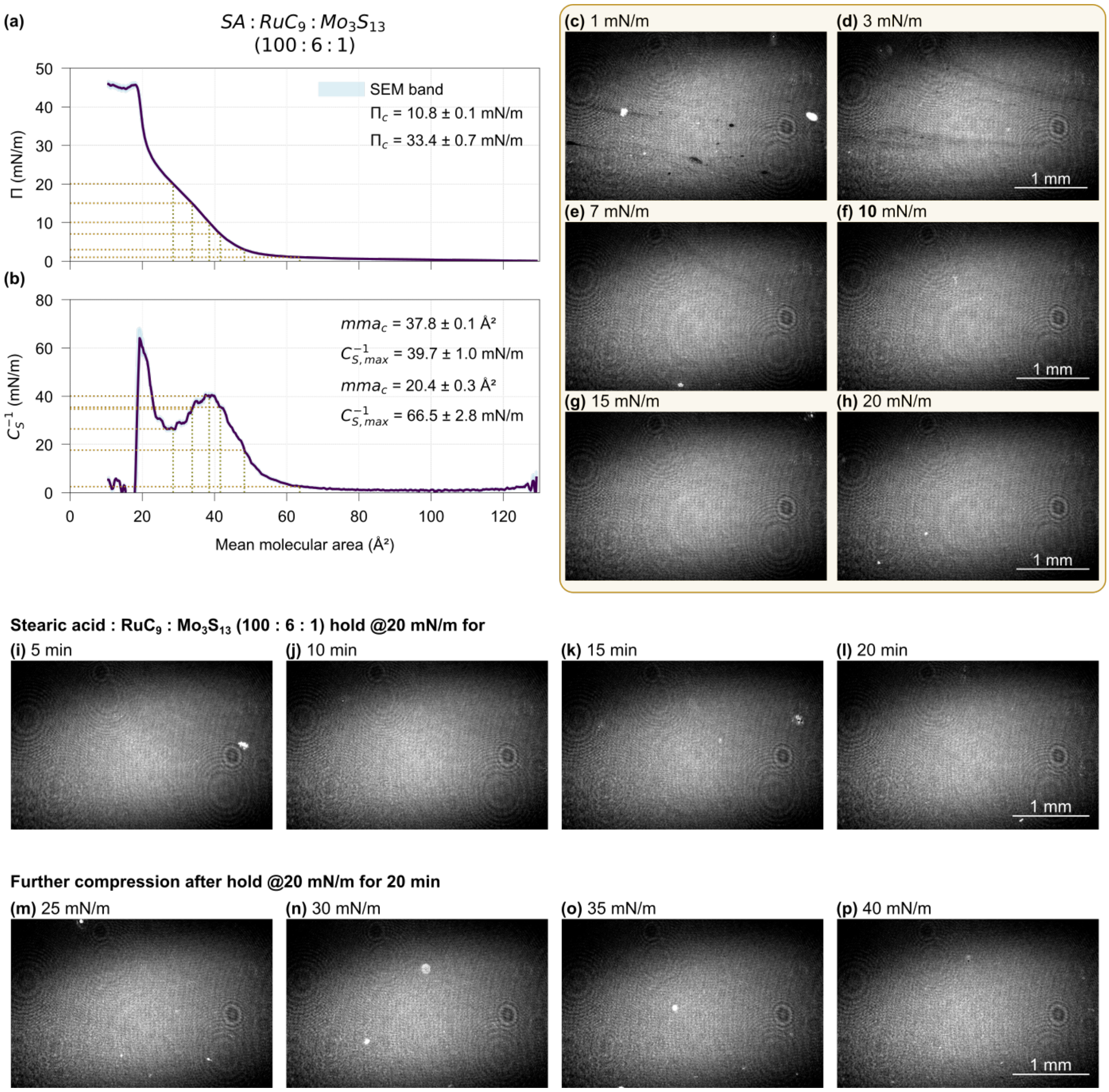

Figure S4: Π(mma) isotherm analysis of **Stearic acid (SA)**:**RuC₉:[Mo₃S₁₃]²⁻** mixture with molar mixing ratio [%] **100:6:1** in (a) and (b). Curves represent the mean of three individual Π(mma) isotherm recordings with the standard error of the mean (SEM) indicated as a band. (c) to (h) BAM images of the course of the Π(mma) isotherm up to 20 mN/m. (i) to (l) hold at 20 mN/m for different time duration. (m) to (t) BAM images of further compression of the Langmuir film after duration of hold at 20 mN/m.

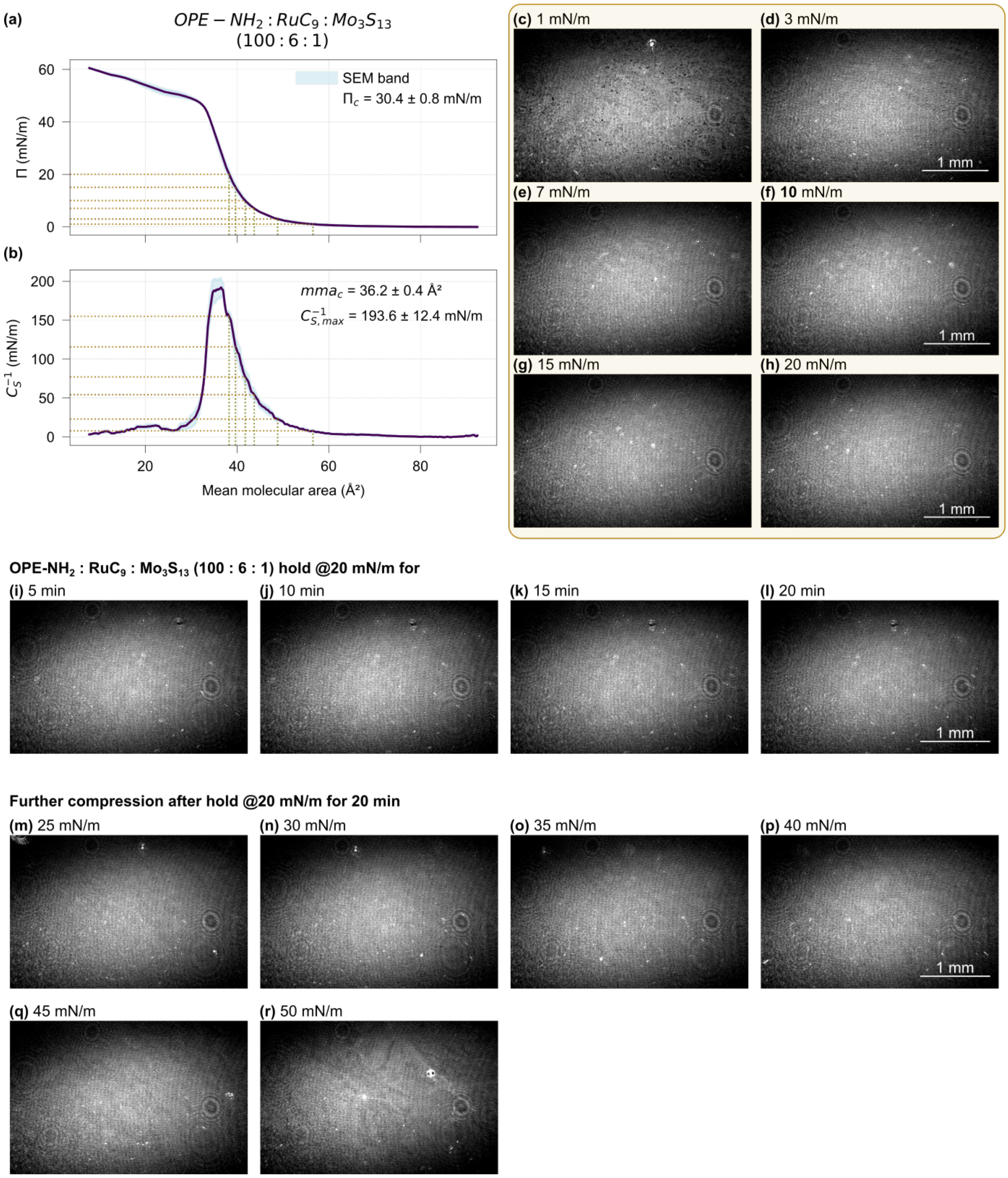

Figure S5: Π(mma) isotherm analysis of **OPE-NH$_2$:RuC$_9$:Mo$_3$S$_{13}$** mixture with molar mixing ratio [%] **100:6:1** in (a) and (b). Curves represent the mean of three individual Π(mma) isotherm recordings with the standard error of the mean (SEM) indicated as a band. (c) to (h) BAM images of the course of the Π(mma) isotherm up to 20 mN/m. (i) to (l) hold at 20 mN/m for different time duration. (m) to (t) BAM images of further compression of the Langmuir film after duration of hold at 20 mN/m.

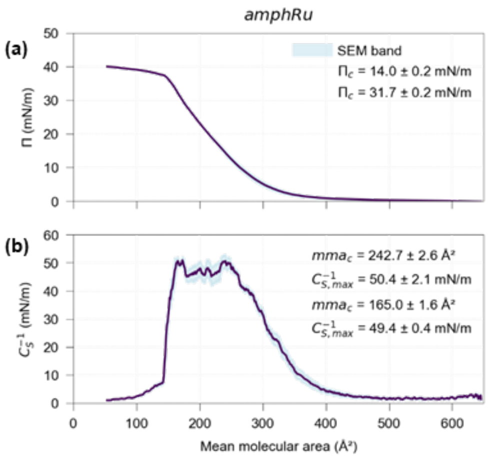

Figure S6: Π(mma) isotherm analysis of **amphRu** in (a) and (b)**.** Curves represent the mean of three individual Π(mma) isotherm recordings with the standard error of the mean (SEM) indicated as a band.

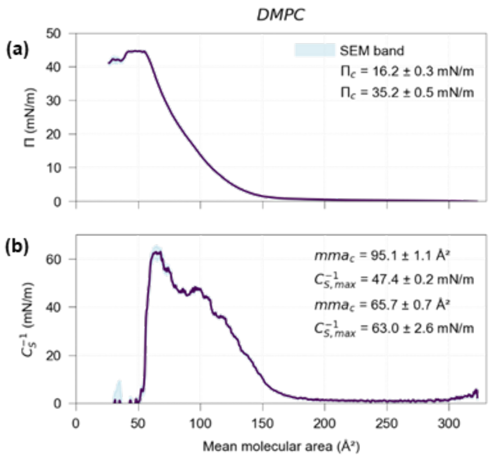

Figure S7: Π(mma) isotherm analysis of **DMPC** in (a) and (b)**.** Curves represent the mean of three individual Π(mma) isotherm recordings with the standard error of the mean (SEM) indicated as a band.

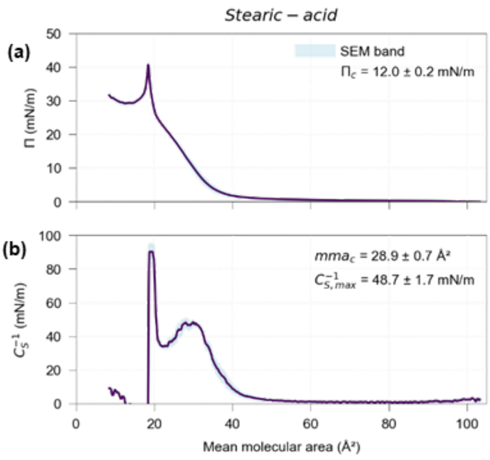

Figure S8: Π(mma) isotherm analysis of **SA** in (a) and (b)**.** Curves represent the mean of three individual Π(mma) isotherm recordings with the standard error of the mean (SEM) indicated as a band.

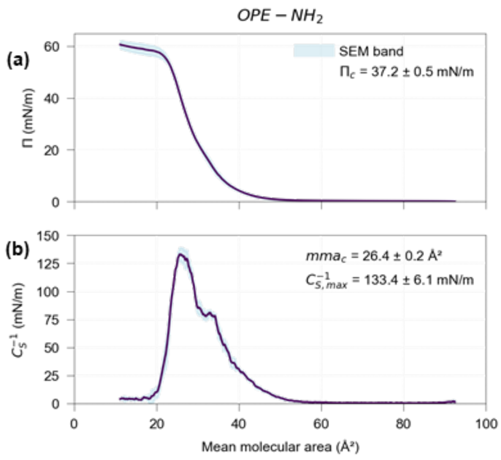

Figure S9: Π(mma) isotherm analysis of **OPE-NH_2** in (a) and (b)**.** Curves represent the mean of three individual Π(mma) isotherm recordings with the standard error of the mean (SEM) indicated as a band.

2.5. Individual Π(mma) isotherm and surface compressional modulus analysis

The characterization of Langmuir monolayer formation was accomplished through the meticulous recording of surface pressure (Π) *vs.* mean molecular area (mma) isotherms. Three to four individual runs were performed. The surface compressional modulus (C_s^{-1}) was calculated from the Π(mma) isotherms.[17] The maximum of the Cs^{-1} curve was identified in order to ascertain the steepest slope in the Π(mma) isotherm. The mean molecular area at the Cs^{-1} maximum is denoted as mma_C, and the corresponding surface pressure at the steepest slope is denoted as Π_C. The results of the individual Π(mma) isotherm recordings are displayed below and on the following pages. The data points used to determine the C_s^{-1} maximum – and, thus, the steepest slope of the Π(mma) isotherm - are highlighted.

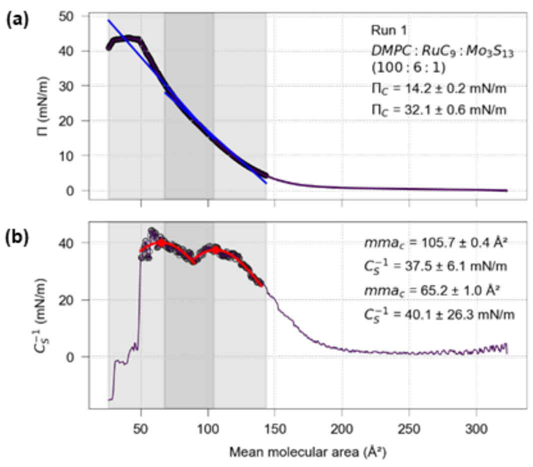

Figure S10: Analysis of **DMPC**:**RuC₉:** **[Mo₃S₁₃]²⁻** Langmuir monolayer formation, **run 1**, with molar mixing ratio (%) **100:6:1**. The upper panel (a) shows the surface pressure (Π) versus mean molecular area (mma) isotherm, and the lower panel (b) displays the surface compressional modulus (Cs^{-1}).

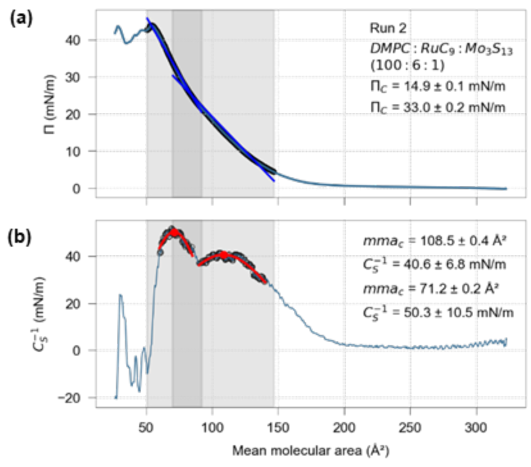

Figure S11: Analysis of **DMPC**: **RuC₉:** **[Mo₃S₁₃]²⁻** Langmuir monolayer formation, **run 2**, with molar mixing ratio (%) **100:6:1**. The upper panel (a) shows the surface pressure (Π) versus mean molecular area (mma) isotherm, and the lower panel (b) displays the surface compressional modulus (Cs^{-1}).

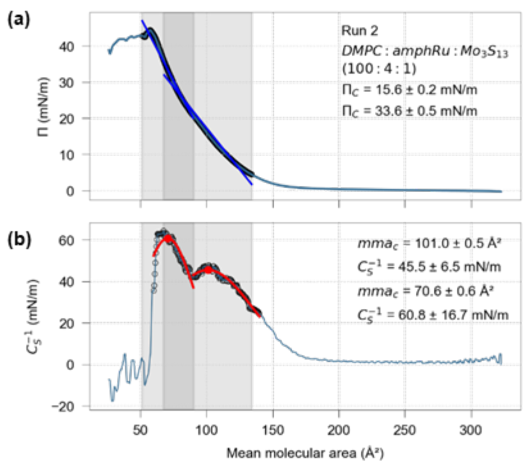

Figure S12: Analysis of **DMPC**:**amphRu:** **[Mo_3S_{13}]$^{2-}$** Langmuir monolayer formation, **run 2**, with molar mixing ratio (%) **100:4:1**. The upper panel (a) shows the surface pressure (Π) versus mean molecular area (mma) isotherm, and the lower panel (b) displays the surface compressional modulus (C_S^{-1}).

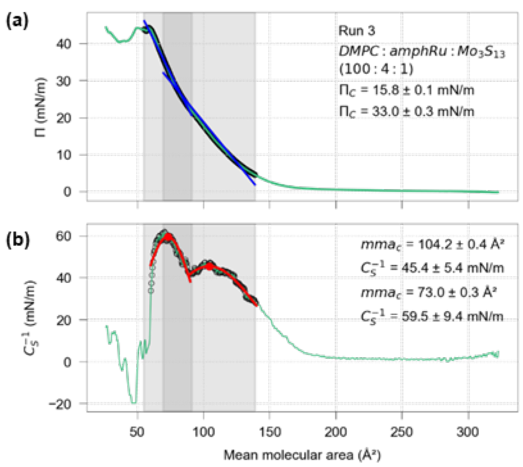

Figure S13: Analysis of **DMPC**:**amphRu:** **[Mo_3S_{13}]$^{2-}$** Langmuir monolayer formation, **run 3**, with molar mixing ratio (%) **100:4:1**. The upper panel (a) shows the surface pressure (Π) versus mean molecular area (mma) isotherm, and the lower panel (b) displays the surface compressional modulus (C_S^{-1}).

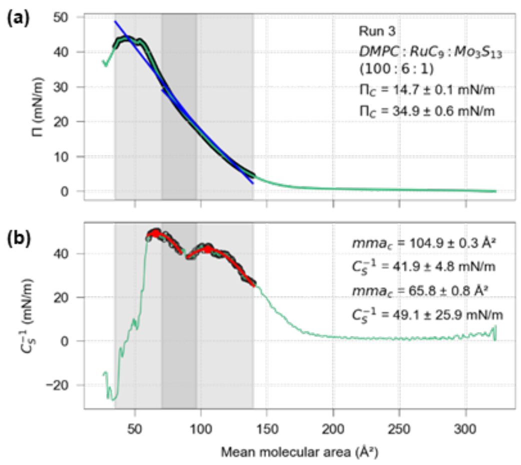

Figure S14: Analysis of **DMPC**:**RuC_9:** **[Mo_3S_{13}]$^{2-}$** Langmuir monolayer formation, **run 3**, with molar mixing ratio (%) **100:6:1**. The upper panel (a) shows the surface pressure (Π) versus mean molecular area (mma) isotherm, and the lower panel (b) displays the surface compressional modulus (C_S^{-1}).

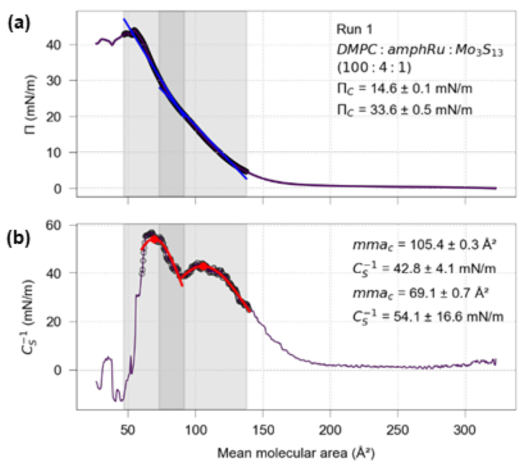

Figure S15: Analysis of **DMPC**:**amphRu:** **[Mo_3S_{13}]$^{2-}$** Langmuir monolayer formation, **run 1**, with molar mixing ratio (%) **100:4:1**. The upper panel (a) shows the surface pressure (Π) versus mean molecular area (mma) isotherm, and the lower panel (b) displays the surface compressional modulus (C_S^{-1}).

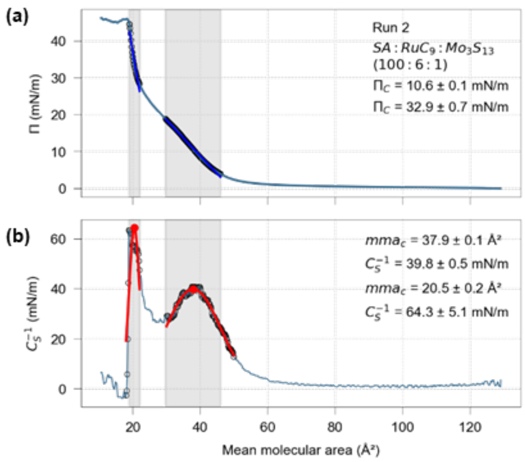

Figure S16: Analysis of **SA**:**RuC$_9$:[Mo$_3$S$_{13}$]$^{2-}$** Langmuir monolayer formation, **run 2**, with molar mixing ratio (%) **100:6:1**. The upper panel (a) shows the surface pressure (Π) versus mean molecular area (mma) isotherm, and the lower panel (b) displays the surface compressional modulus (Cs^{-1}).

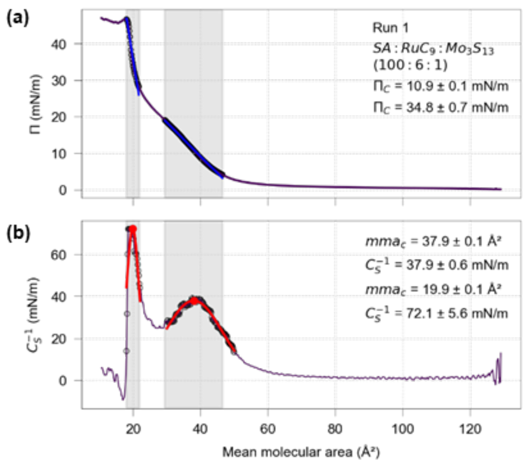

Figure S17: Analysis of **SA**:**RuC$_9$: [Mo$_3$S$_{13}$]$^{2-}$** Langmuir monolayer formation, **run 1**, with molar mixing ratio (%) **100:6:1**. The upper panel (a) shows the surface pressure (Π) versus mean molecular area (mma) isotherm, and the lower panel (b) displays the surface compressional modulus (Cs^{-1}).

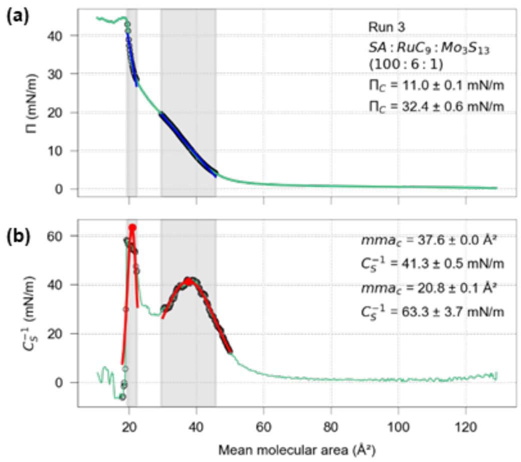

Figure S18: Analysis of **SA**:**RuC$_9$: [Mo$_3$S$_{13}$]$^{2-}$** Langmuir monolayer formation, **run 3**, with molar mixing ratio (%) **100:6:1**. The upper panel (a) shows the surface pressure (Π) versus mean molecular area (mma) isotherm, and the lower panel (b) displays the surface compressional modulus (Cs^{-1}).

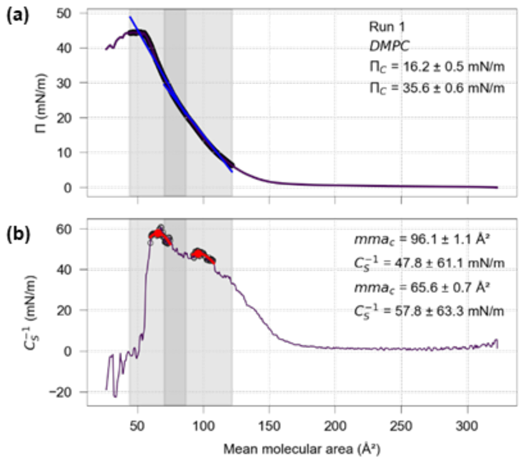

Figure S19: Analysis of **DMPC** Langmuir monolayer formation, **run 1**. The upper panel (a) shows the surface pressure (Π) versus mean molecular area (mma) isotherm, and the lower panel (b) displays the surface compressional modulus (Cs^{-1}).

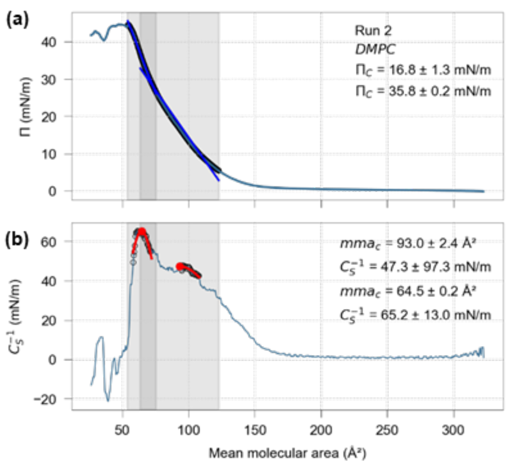

Figure S20: Analysis of **DMPC** Langmuir monolayer formation, **run 2**. The upper panel (a) shows the surface pressure (Π) versus mean molecular area (mma) isotherm, and the lower panel (b) displays the surface compressional modulus (Cs^{-1}).

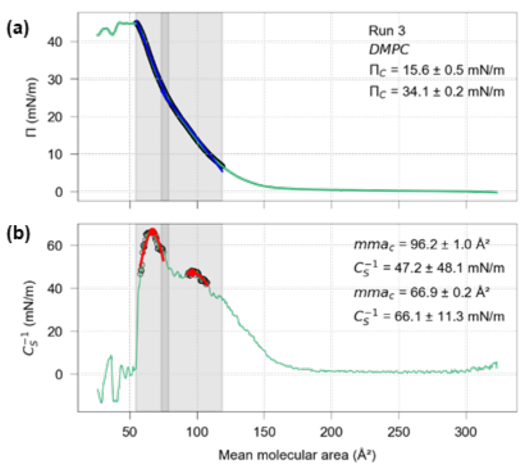

Figure S21: Analysis of **DMPC** Langmuir monolayer formation, **run 3**. The upper panel (a) shows the surface pressure (Π) versus mean molecular area (mma) isotherm, and the lower panel (b) displays the surface compressional modulus (Cs^{-1}).

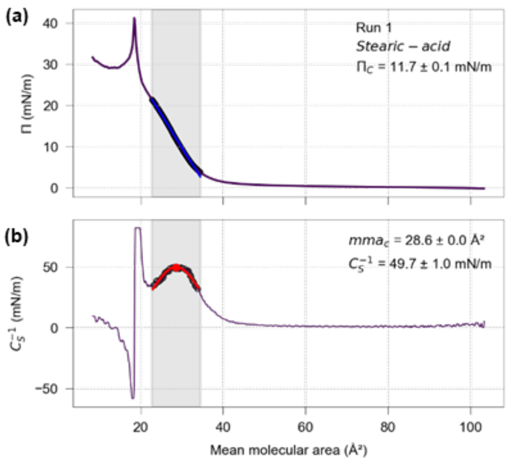

Figure S22: Analysis of **SA** Langmuir monolayer formation, **run 1**. The upper panel (a) shows the surface pressure (Π) versus mean molecular area (mma) isotherm, and the lower panel (b) displays the surface compressional modulus (Cs^{-1}).

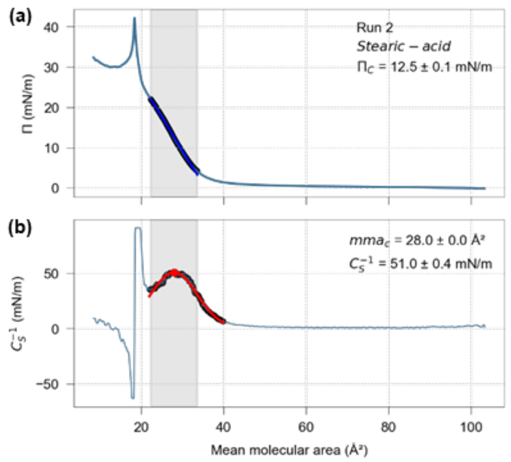

Figure S23: Analysis of **SA** Langmuir monolayer formation, **run 2**. The upper panel (a) shows the surface pressure (Π) versus mean molecular area (mma) isotherm, and the lower panel (b) displays the surface compressional modulus (Cs^{-1}).

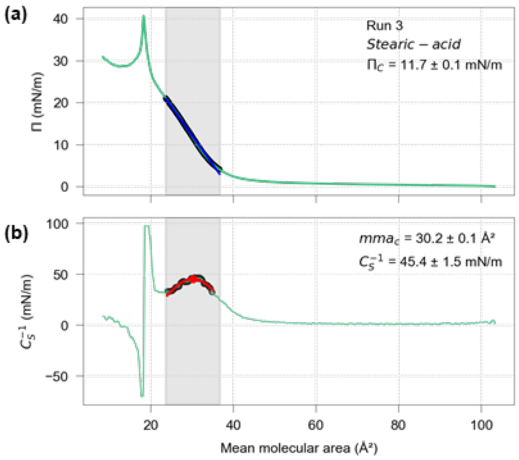

Figure S24: Analysis of **SA** Langmuir monolayer formation, **run 3**. The upper panel (a) shows the surface pressure (Π) versus mean molecular area (mma) isotherm, and the lower panel (b) displays the surface compressional modulus (Cs^{-1}).

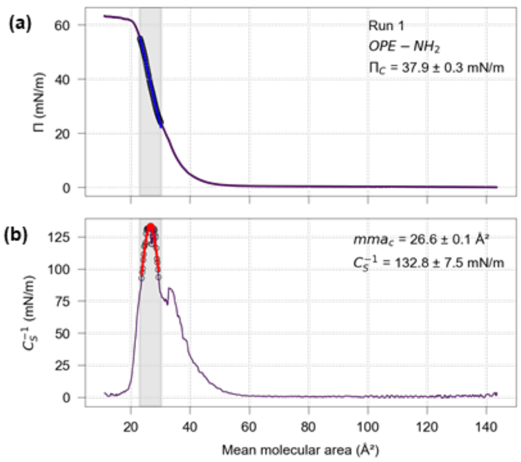

Figure S25: Analysis of **OPE-NH₂** Langmuir monolayer formation, **run 1**. The upper panel (a) shows the surface pressure (Π) versus mean molecular area (mma) isotherm, and the lower panel (b) displays the surface compressional modulus (Cs^{-1}).

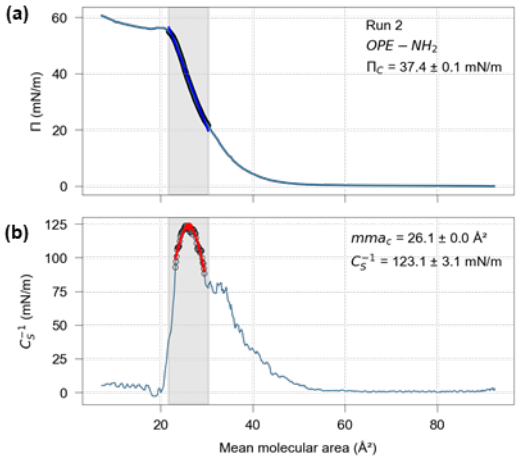

Figure S26: Analysis of **OPE-NH₂** Langmuir monolayer formation, **run 2**. The upper panel (a) shows the surface pressure (Π) versus mean molecular area (mma) isotherm, and the lower panel (b) displays the surface compressional modulus (Cs^{-1}).

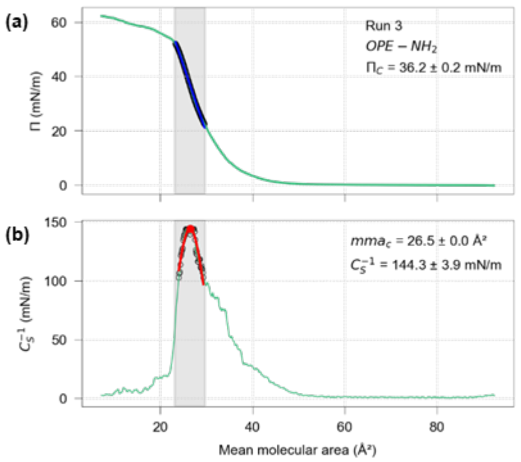

Figure S27: Analysis of **OPE-NH₂** Langmuir monolayer formation, **run 3**. The upper panel (a) shows the surface pressure (Π) versus mean molecular area (mma) isotherm, and the lower panel (b) displays the surface compressional modulus (Cs^{-1}).

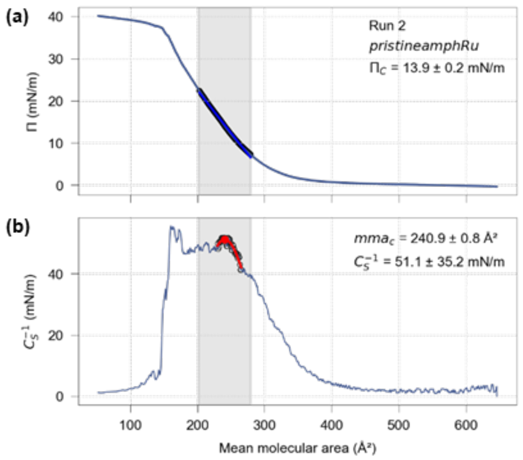

Figure S28: Analysis of **amphRu** Langmuir monolayer formation, **run 2**. The upper panel (a) shows the surface pressure (Π) versus mean molecular area (mma) isotherm, and the lower panel (b) displays the surface compressional modulus (Cs^{-1}).

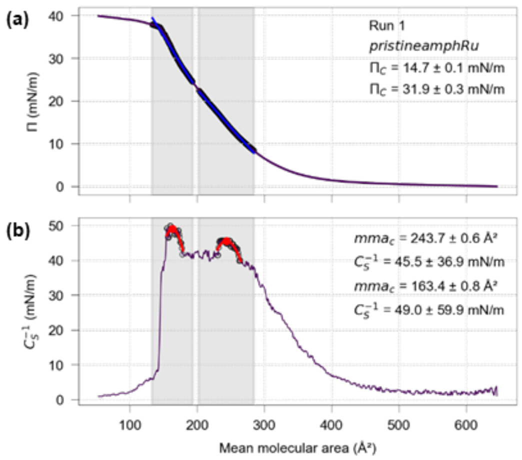

Figure S29: Analysis of **amphRu** Langmuir monolayer formation, **run 1**. The upper panel (a) shows the surface pressure (Π) versus mean molecular area (mma) isotherm, and the lower panel (b) displays the surface compressional modulus (Cs^{-1}).

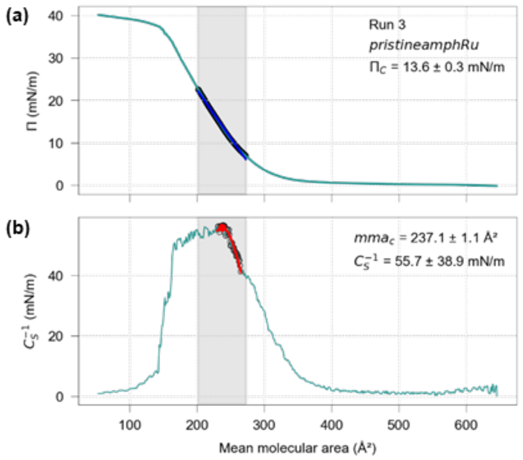

Figure S30: Analysis of **amphRu** Langmuir monolayer formation, **run 3**. The upper panel (a) shows the surface pressure (Π) versus mean molecular area (mma) isotherm, and the lower panel (b) displays the surface compressional modulus (Cs^{-1}).

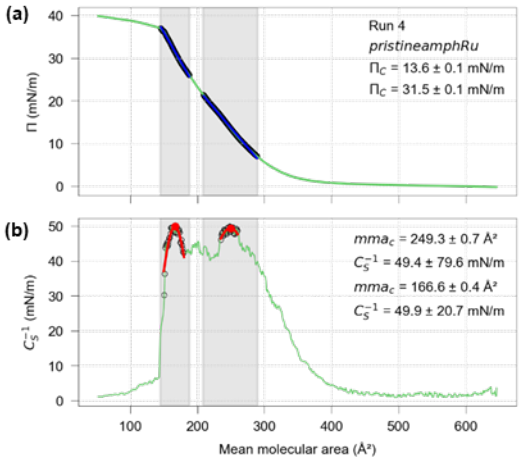

Figure S31: Analysis of **amphRu** Langmuir monolayer formation, **run 4**. The upper panel (a) shows the surface pressure (Π) versus mean molecular area (mma) isotherm, and the lower panel (b) displays the surface compressional modulus (Cs^{-1}).

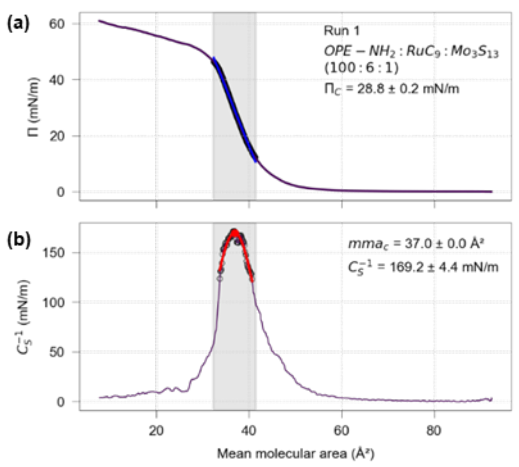

Figure S32: Analysis of **OPE-NH$_2$:RuC$_9$:[Mo$_3$S$_{13}$]$^{2-}$** Langmuir monolayer formation, **run 1**, with molar mixing ratio (%) **100:6:1**. The upper panel (a) shows the surface pressure (Π) versus mean molecular area (mma) isotherm, and the lower panel (b) displays the surface compressional modulus (C_s^{-1}).

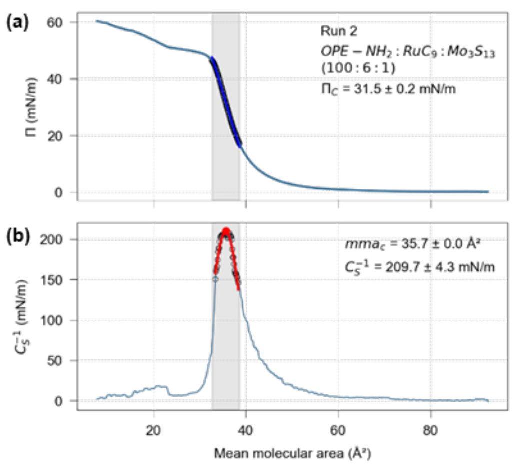

Figure S33: Analysis of **OPE-NH$_2$:RuC$_9$:[Mo$_3$S$_{13}$]$^{2-}$** Langmuir monolayer formation, **run 2**, with molar mixing ratio (%) **100:6:1**. The upper panel (a) shows the surface pressure (Π) versus mean molecular area (mma) isotherm, and the lower panel (b) displays the surface compressional modulus (C_s^{-1}).

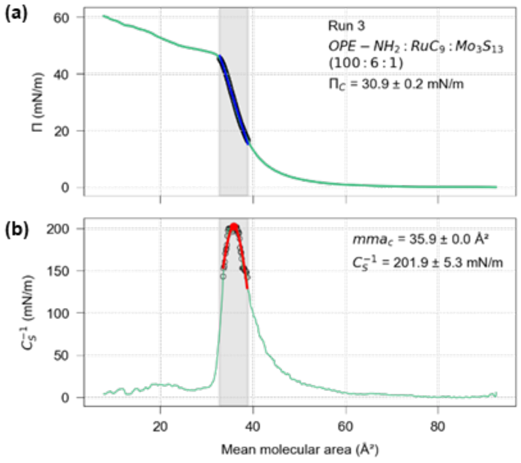

Figure S34: Analysis of **OPE-NH$_2$:RuC$_9$:[Mo$_3$S$_{13}$]$^{2-}$** Langmuir monolayer formation, **run 3**, with molar mixing ratio (%) **100:6:1**. The upper panel (a) shows the surface pressure (Π) versus mean molecular area (mma) isotherm, and the lower panel (b) displays the surface compressional modulus (C_s^{-1}).

3. Total reflection X-Ray Fluorescence (TXRF) analysis

TXRF measurements were performed using high-efficiency module S2 Picofox and S4 T-STAR (Bruker Nano GmbH, Berlin, Germany) equipped with molybdenum and tungsten X-ray, respectively. The samples were excited at maximum power conditions (Mo: 50 kV and 600 µA; W: 50 kV and 1000 µA) and measured using a live time of 1000 s. **DMPC**- and **OPE-NH$_2$**-based-LS and -LB samples were deposited on pre-cleaned, non-siliconized quartz glass sample carriers. To ensure film integrity and to prevent damage caused by the TXRF measuring cassette, a self-designed adhesive film (silver, Cricut, Inc., South Jordan, UT, USA) cut using a plotter with a circular cutout (1 cm diameter) in the middle, was attached onto the sample carrier prior to sample preparation. The adhesive film was removed before TXRF measurement. All prepared sample carriers, as well as a sample carrier without sample, were measured in total nine times with a rotation by 45° after each measurement.

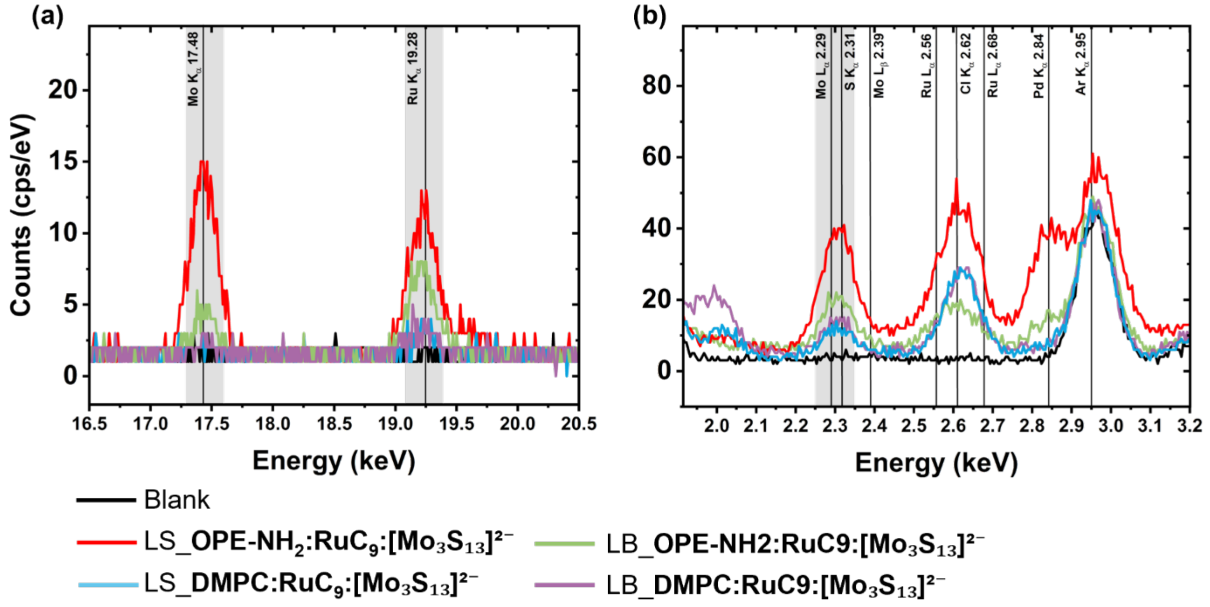

Figure S35 Averaged TXRF spectra (n = 9) of **DMPC**- and **OPE-NH$_2$**-based-LS and -LB films on TXRF sample carrier and blank sample carrier (black) in the energy range from (a) 16.5 to 20.5 keV (S4 T-STAR) and (b) 1.9 to 3.2 keV (S2 Picofox). X-ray fluorescence energies of detected elements are marked by vertical black lines. The regions of interest (ROI; 2.25 to 2.35 keV) including sulphur (S K$_\alpha$ = 2.31 keV) and molybdenum (Mo L$_\alpha$ = 2.29 keV) energy lines (a) as well as (ROI; 17.35 to 17.55 keV) molybdenum (Mo K$_\alpha$ = 17.48 keV) and (ROI; 19.10 to 19.30 keV) ruthenium (Ru K$_\alpha$ = 19.28 keV) energy lines (b) are indicated by grey shades. LS_**OPE-NH$_2$**:**RuC$_9$:[Mo$_3$S$_{13}$]$^{2-}$** (red curve); LS_**DMPC**:**RuC$_9$:[Mo$_3$S$_{13}$]$^{2-}$** (blue curve); LB_**OPE-NH$_2$**:**RuC$_9$:[Mo$_3$S$_{13}$]$^{2-}$** (green curve); LB_**DMPC**:**RuC$_9$:[Mo$_3$S$_{13}$]$^{2-}$** (violet curve); blank sample (black curve).

Table S1. Mean signal with standard deviation (± 1 SD; n = 21) in the regions of interest. Limit of detection (LOD) is calculated using 3σ criterium. (Mean signals below the LOD are given in italics.)

Region of interest	*Mo L_α* (2.25 – 2.35 keV)	*Mo K_α* (17.35 – 17.55 keV)	*Ru K_α* (19.10 – 19.30 keV)
LOD / cps	6.6	3.6	2.9
Sample		*Mean ± SD / cps*	
Blank	3.9 ± 0.9	1.7 ± 0.7	1.4 ± 0.5
LS_**OPE-NH_2:RuC_9:[Mo_3S_{13}]$^{2-}$**	33.1 ± 6.1	12.6 ± 1.8	10.1 ± 1.7
LS_**DMPC:RuC_9:[Mo_3S_{13}]$^{2-}$**	10.2 ± 1.9	*1.9 ± 0.5*	3.0 ± 0.8
LB_**OPE-NH_2:RuC_9:[Mo_3S_{13}]$^{2-}$**	17.6 ± 2.8	4.1 ± 0.8	6.7 ± 1.2
LB_**DMPC:RuC_9:[Mo_3S_{13}]$^{2-}$**	12.0 ± 2.3	*2.1 ± 0.6*	3.1 ± 0.9

4. X-ray Photoelectron Spectroscopy (XPS) analysis

High-resolution XP spectra were acquired to confirm the incorporation of the photosensitizer (**amphRu**) and the **DMPC** as well as the **OPE-NH_2** matrix within the Langmuir films. XPS was conducted using a K-alpha spectrometer (ThermoScientific) with a monochromatic X-ray source (Al K_α) with a spot diameter of 400 µm and an electron detector with 0.5 eV energy resolution. An internal flood gun was employed for charge compensation. The spectra were calibrated using the Si 2p peak (103.5 eV) and fitted using Voigt functions after background subtraction. As the Ru $3d_{3/2}$ peak is covered by the C 1s species, the Ru 3d doublet was fitted with a binding energy distance between Ru $3d_{5/2}$ and $3d_{3/2}$ of 4.2 eV, an intensity ratio of 3 : 2 and the same fwhm.

Fig. S36 shows XP spectra of Langmuir-Blodget (LB, left) and Langmuir-Schaefer (LS, right) deposited **DMPC:amphRu:[Mo_3S_{13}]$^{2-}$** samples with a molar mixing ratio (%) 100:4:1 on glass substrates. Even though the Ru 3d region overlaps with the C 1s envelope a distinct Ru $3d_{5/2}$ contribution is observed at a binding energy (BE) of ~281.8 eV, consistent with Ru(II) in polypyridyl complexes (**Fig. S36 c,d**). The C 1s spectra of the **DMPC:amphRu:[Mo_3S_{13}]$^{2-}$** films (both LS and LB configurations) display the expected dominant C–C/C–H component, accompanied by C–N/C–O (~286 eV) and carboxylate (~288–289 eV) shoulders, in agreement with the chemical groups of DMPC matrix and the photosensitizer. In the LS-transferred films containing stearic acid (**Fig. S36d**), an additional intense aliphatic C–C component is observed, which alters the overall line shape. This LS sample also exhibit moderate surface charging, likely caused by the long-chain fatty acid layer, resulting in a slight positive shift of all core-level binding energies. The N 1s spectra (**Fig. S36 e,f**) show two chemically distinct environments: a component at a BE of ~403.2 eV, assigned to the quaternary ammonium (N^+) site of DMPC and a component at a BE of ~400.7 eV, attributed to the bipyridyl nitrogen atoms of the Ru-photosensitizer. Measurements performed on films deposited onto Si substrates (**Fig. S37**) yielded

spectra with significantly improved signal-to-noise ratios and narrower peaks. The same chemical features were detected, confirming reproducibility across substrates. The absence of stearic acid in these samples results in a cleaner C 1s envelope without charging-related distortions.

Fig. S38 shows XP spectra of Langmuir-Blodget (LB, left) and Langmuir-Schaefer (LS, right) deposited **OPE-NH$_2$:amphRu:[Mo$_3$S$_{13}$]$^{2-}$** films with a molar mixing ratio (%) 100:4:1 on glass substrates. The C 1s spectra show similar spectral features as for the **DMPC** matrix confirming the successful detection of the **OPE-NH$_2$** matrix. The photosensitizer is again visible as a distinct Ru 3d$_{5/2}$ contribution observed at a BE of ~281.8 eV. In the N 1s spectra, clear differences between the **DMPC** and the **OPE-NH$_2$** matrices are visible. Instead of the peak assigned to the N$^+$ site of the DMPC, the NH$_2$ bonds of the **OPE-NH$_2$** are detected via the intense peak at a BE of ~399.2 eV. Furthermore, the photosensitizer is again visible with a small N$_{bpy}$ peak at a BE of ~400.7 eV. This confirms both the presence of photosensitizer and matrix. Similarly to the **DMPC** matrix discussed before, measurements performed on films deposited onto Si substrates (**Fig. S39**) yielded spectra with significantly improved signal-to-noise ratios and narrower peaks detecting the same chemical features. Moreover, the higher quality of the C 1s spectra (both LS and LB configurations) show an additional peak at a BE of ~ 283.8 eV assigned to the C≡C bond of the **OPE-NH$_2$** matrices. This is further confirming the successful detection of both matrices.

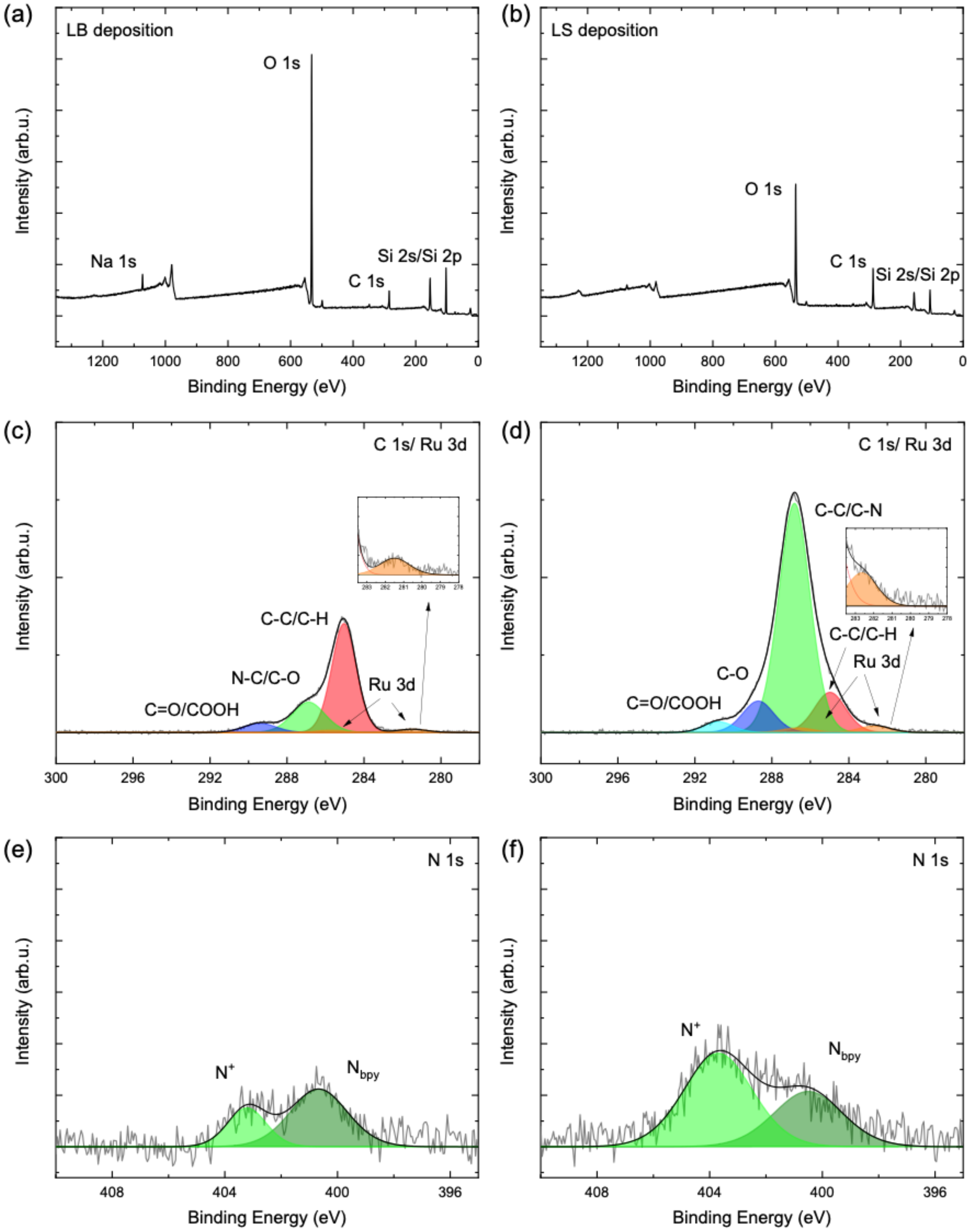

Figure S36. X-ray photoelectron (XPS) spectra of (a) Langmuir-Blodget (LB) and (b) Langmuir-Schaefer (LS) **DMPC:amphRu:[Mo_3S_{13}]$^{2-}$** with a molar mixing ratio (%) **100:4:1** deposited on glass substrates. (a, b) The overview spectrum and (c, d) the high-resolution C 1s/Ru 3d and (e,f) N 1s spectra are shown.

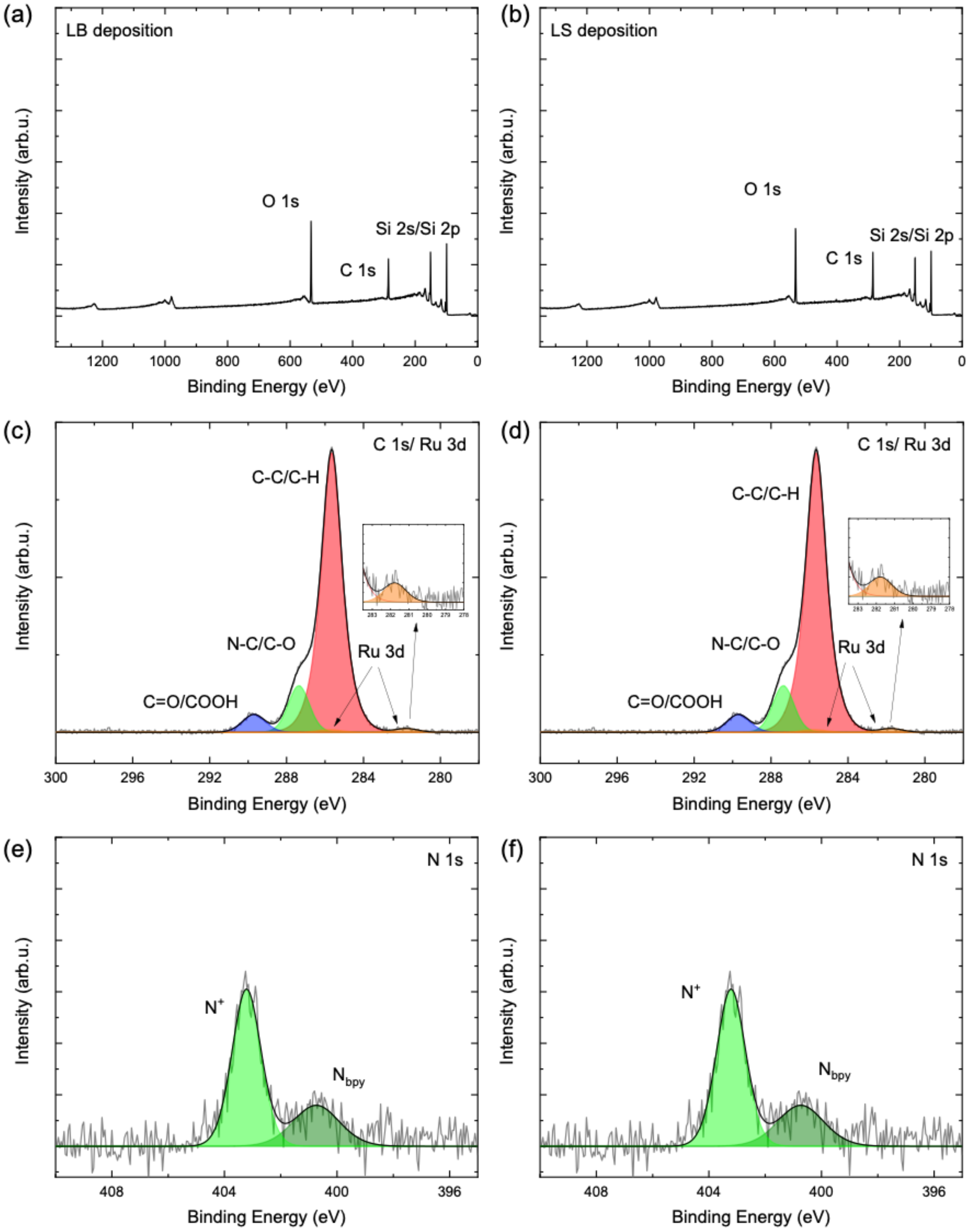

Figure S37. X-ray photoelectron (XPS) spectra of (a) Langmuir-Blodget (LB) and (b) Langmuir-Schaefer (LS) **DMPC:amphRu:[Mo_3S_{13}]$^{2-}$** with a molar mixing ratio (%) **100:4:1** deposited on silicon substrates. (a, b) The overview spectrum and (c, d) the high-resolution C 1s/Ru 3d and (e,f) N 1s spectra are shown.

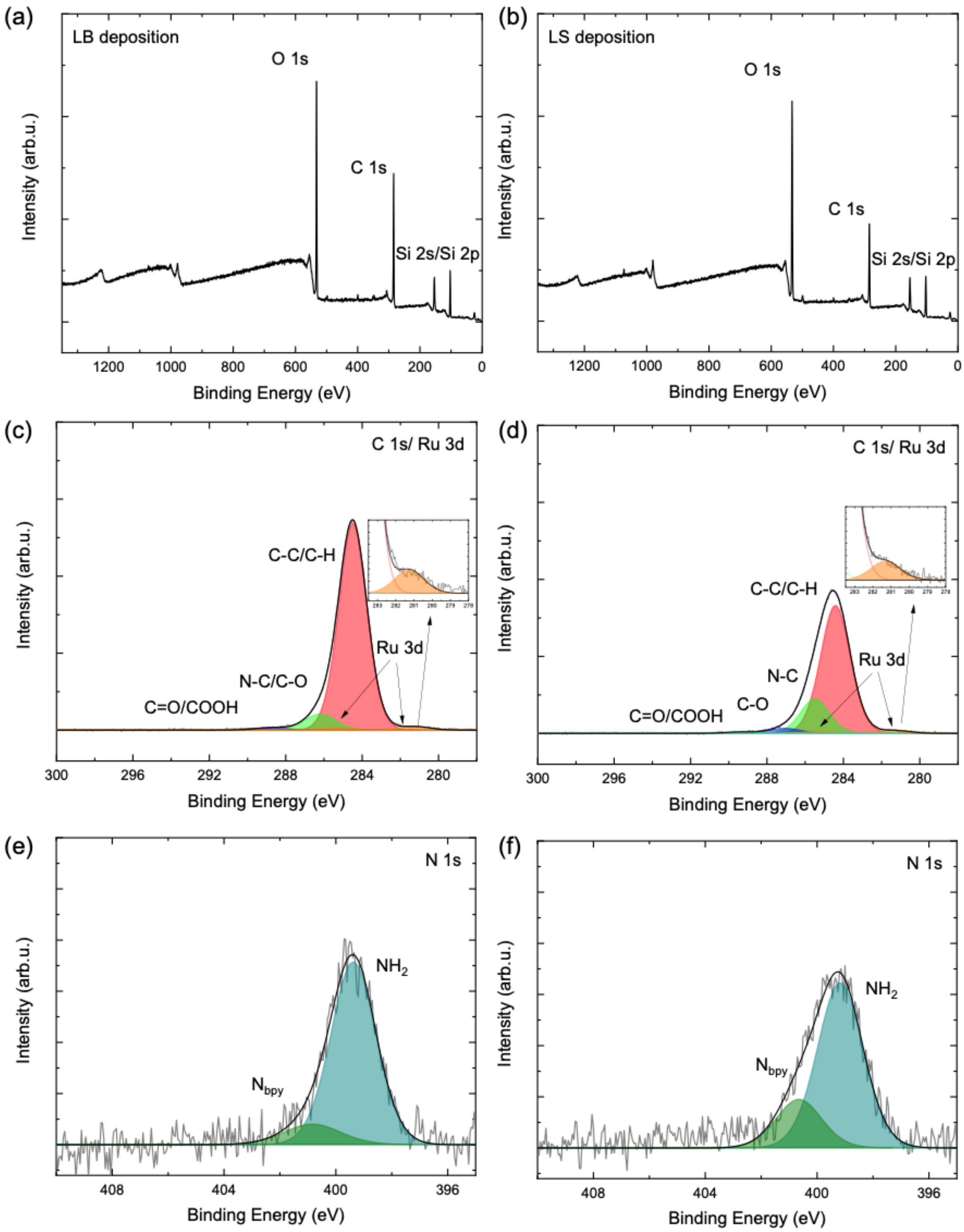

Figure S38. X-ray photoelectron (XPS) spectra of (a) Langmuir-Blodget (LB) and (b) Langmuir-Schaefer (LS) **OPE-NH$_2$:amphRu:[Mo$_3$S$_{13}$]$^{2-}$** with a molar mixing ratio (%) 100:4:1 deposited on glass substrates. (a, b) The overview spectrum and (c, d) the high-resolution C 1s/Ru 3d and (e,f) N 1s spectra are shown.

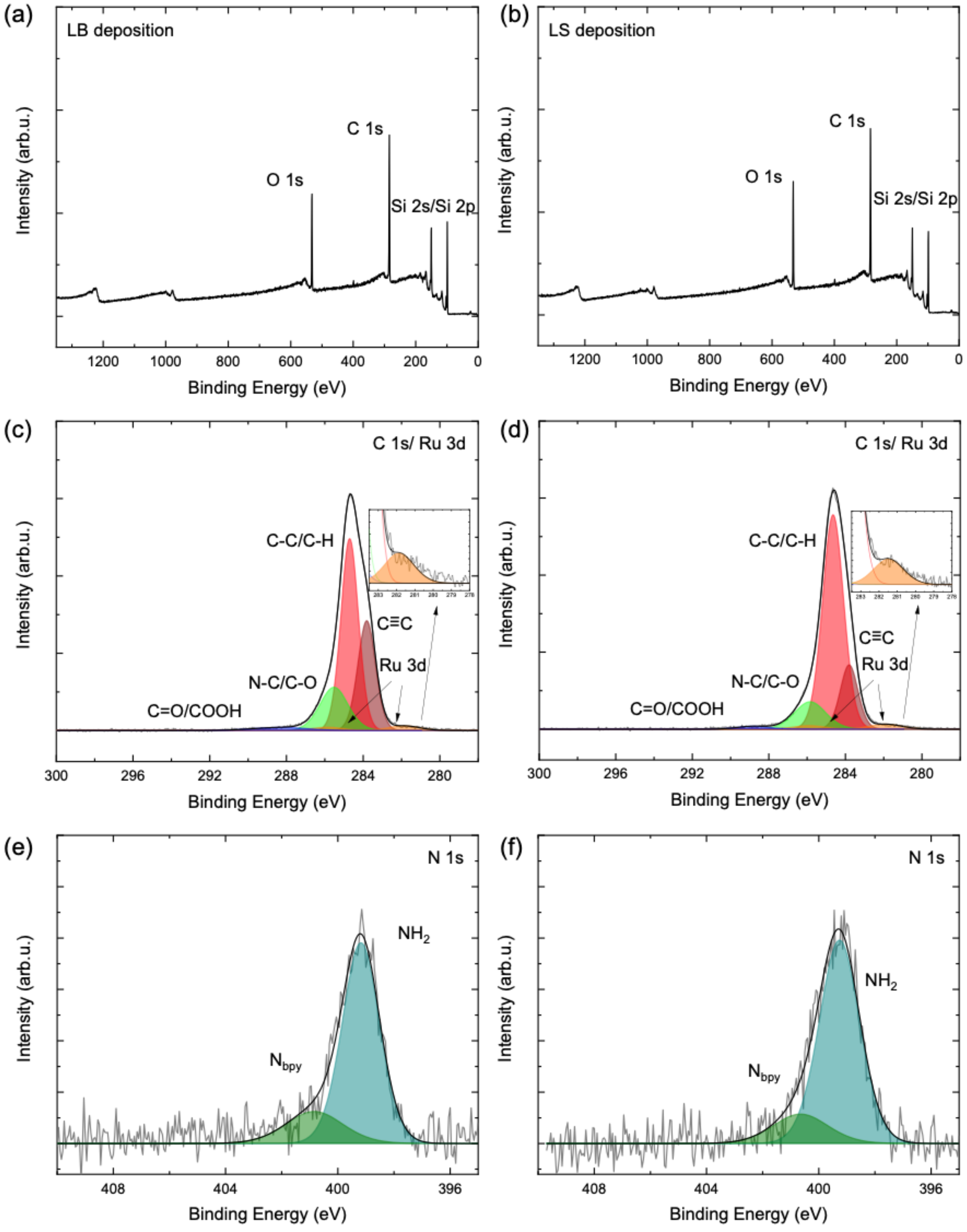

Figure S39. X-ray photoelectron (XPS) spectra of (a) Langmuir-Blodget (LB) and (b) Langmuir-Schaefer (LS) **OPE-NH$_2$:amphRu:[Mo$_3$S$_{13}$]$^{2-}$** with a molar mixing ratio (%) 100:4:1 deposited on silicon substrates. (a, b) The overview spectrum and (c, d) the high-resolution C 1s/Ru 3d and (e,f) N 1s spectra are shown.

5. Hydrogen evolution *via* chronoamperometry

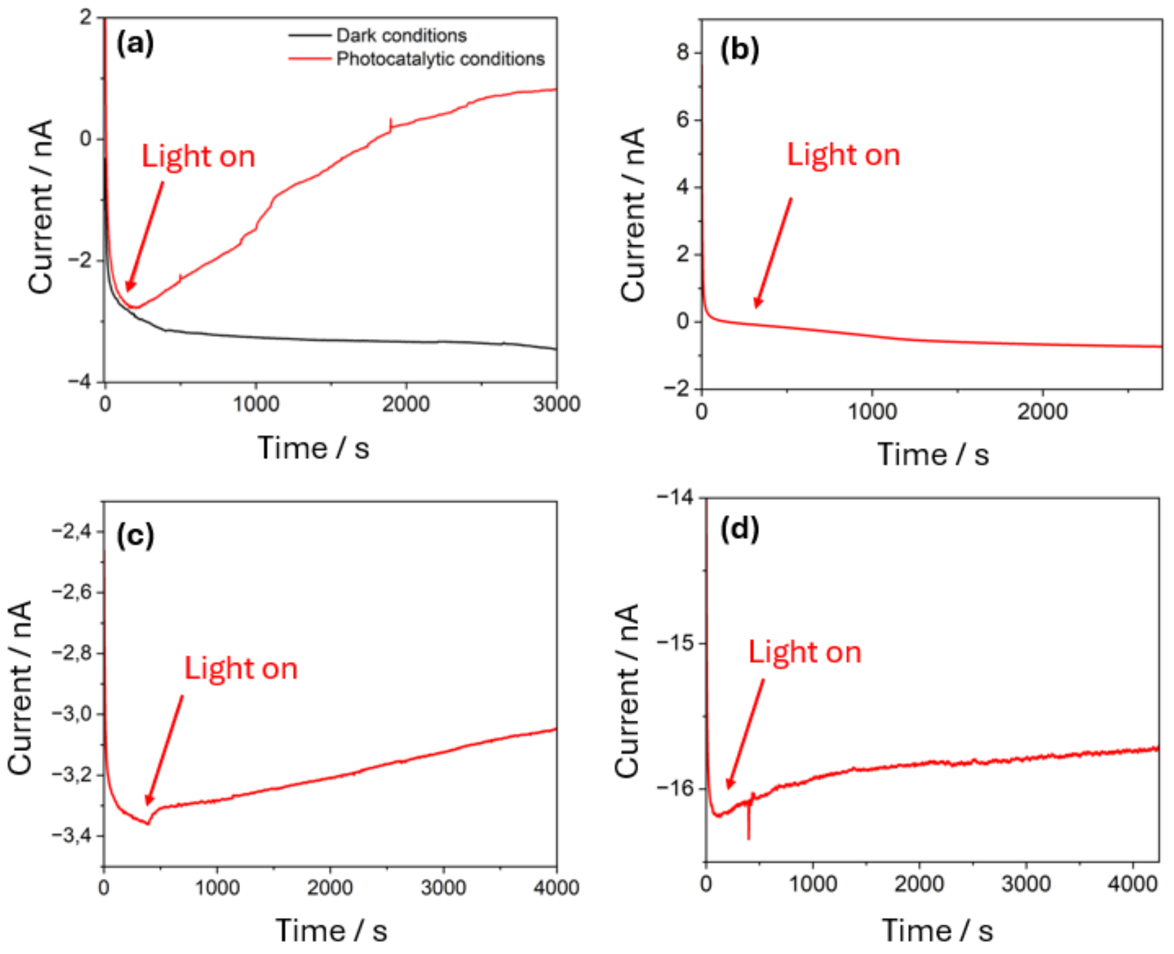

Figure S40. Exemplary chronoamperometry curves recorded under illumination (red line) and in dark conditions (black line) in ascorbic acid using a Pt-B microsensor. SECM probe-substrate distance is 20 µm. E_{probe} = -0.05 V *vs* Ag/AgCl. a) Langmuir-Blodgett film with PS = **RuC_9**, Matrix: **DMPC,** and **$[Mo_3S_{13}]^{2-}$** as CAT; b) Langmuir-Schaefer film with PS = **AmphRu**, Matrix: **DMPC**, and **$[Mo_3S_{13}]^{2-}$** as CAT; c) Langmuir-Blodgett film with PS = **AmphRu**, Matrix: **DMPC**, and **$[Mo_3S_{13}]^{2-}$** as CAT and d) Langmuir-Blodgett film with PS = **RuC_9**, Matrix: **OPE-NH_2** and **$[Mo_3S_{13}]^{2-}$** as CAT.

Table S2: H_2 evolution rates and AQEs of LB and LS films containing RuC_9 and $[Mo_3S_{13}]^{2-}$.

Matrix	H_2 evolution rate / fmol s^{-1} mm^{-2}	AQE	AQE %
OPE-NH_2 (LB) (S1)	938 ± 322	0.0032	0.32
OPE-NH_2 (LB) (S2)	741 ± 227	0.0025	0.25
OPE-NH_2 (LB) (S3)	443 ± 215	0.0015	0.15
OPE-NH_2 (LS) (S1)	0	0.0000	0.00
OPE-NH_2 (LS) (S2)	0	0.0000	0.00

OPE-NH_2 (LS) (S3)	0	0.0000	0.00
DMPC (LS) (S1)	308 ± 57	0.0011	0.11
DMPC (LS) (S2)	430 ± 83	0.0015	0.15
DMPC (LS) (S3)	350 ± 40	0.0012	0.12
DMPC (LB) (S1)	426 ± 101	0.0015	0.15
DMPC (LB) (S2)	348 ± 46	0.0012	0.12
DMPC (LB) (S3)	347 ± 47	0.0012	0.12

Table S3: H_2 evolution rates and AQEs of **DMPC**-based-Langmuir films using **RuC_9 or amphRu** as PS and $[Mo_3S_{13}]^{2-}$ as CAT.

Photosensitizer	H_2 evolution rate / fmol s^{-1} mm^{-2}	AQE	AQE %
RuC_9 (LS)	350 ± 40	0.0012	0.12
RuC_9 (LB)	348 ± 46	0.0012	0.12
AmphRu (LB)	188 ± 17	0.0006	0.06
AmphRu (LS)	0	0.0000	0.00
